# Radiotherapy-activated NBTXR3 nanoparticles promote ferroptosis through induction of lysosomal membrane permeabilization

**DOI:** 10.1186/s13046-023-02938-0

**Published:** 2024-01-03

**Authors:** Jordan Da Silva, Célia Bienassis, Peter Schmitt, Céline Berjaud, Mickael Guedj, Sébastien Paris

**Affiliations:** Nanobiotix, Paris, France

**Keywords:** Radiotherapy, Ferroptosis, Lipid peroxidation, Lysosome membrane permeabilization, Nanoparticle, NBTXR3

## Abstract

**Purpose:**

Radiotherapy-activated NBTXR3 (NBTXR3 + RT) has demonstrated superior efficacy in cancer cell destruction and tumor growth control, compared to radiotherapy (RT), in preclinical and clinical settings. Previous studies highlighted the immunomodulatory properties of NBTXR3 + RT, such as modification of tumor cell immunogenicity/adjuvanticity, producing an effective local tumor control and abscopal effect, related to an enhanced antitumor immune response. Furthermore, NBTXR3 + RT has shown potential in restoring anti-PD1 efficacy in a refractory tumor model. However, the early events leading to these results, such as NBTXR3 endocytosis, intracellular trafficking and primary biological responses induced by NBTXR3 + RT remain poorly understood.

**Methods:**

We analyzed by transmission electron microscopy endocytosis and intracellular localization of NBTXR3 nanoparticles after endocytosis in various cell lines, in vitro and in vivo. A kinetic of NBTXR3 endocytosis and its impact on lysosomes was conducted using LysoTracker staining, and a RNAseq analysis was performed. We investigated the ability of NBTXR3 + RT to induce lysosomal membrane permeabilization (LMP) and ferroptosis by analyzing lipid peroxidation. Additionally, we evaluated the recapture by cancer cells of NBTXR3 released from dead cells.

**Results:**

NBTXR3 nanoparticles were rapidly internalized by cells mainly through macropinocytosis and in a less extend by clathrin-dependent endocytosis. NBTXR3-containing endosomes were then fused with lysosomes. The day following NBTXR3 addition, we measured a significant increase in LysoTracker lysosome labeling intensity, in vitro as in vivo. Following RT, a significant lysosomal membrane permeabilization (LMP) was measured exclusively in cells treated with NBTXR3 + RT, while RT had no effect. The day post-irradiation, a significant increase in lipid peroxidation, a biomarker of ferroptosis, was measured with NBTXR3 + RT compared to RT. Moreover, we demonstrated that NBTXR3 nanoparticles released from dead cells can be recaptured by cancer cells.

**Conclusions:**

Our findings provide novel insights into the early and specific biological effects induced by NBTXR3 + RT, especially LMP, not induced by RT in our models. The subsequent significant increase in lipid peroxidation partially explains the enhanced cancer cell killing capacity of NBTXR3 + RT compared to RT, potentially by promoting ferroptosis. This study improves our understanding of the cellular mechanisms underlying NBTXR3 + RT and highlights its potential as an agnostic therapeutic strategy for solid cancers treatment.

**Graphical Abstract:**

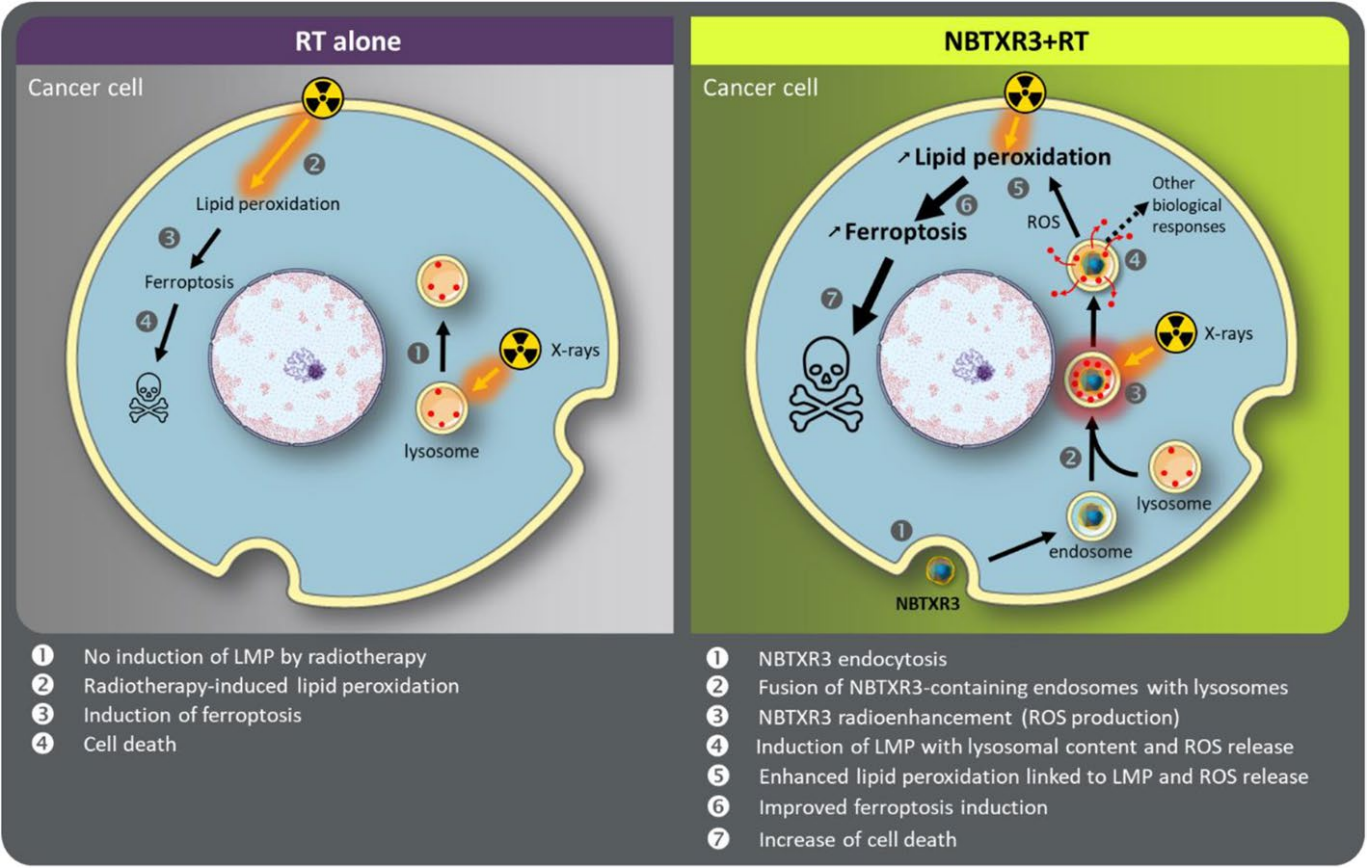

**Supplementary Information:**

The online version contains supplementary material available at 10.1186/s13046-023-02938-0.

## Introduction

For decades, radiotherapy (RT) has been a fundamental component of solid tumor treatment [1, 2]. Currently, at least half of cancer patients undergo radiotherapy as part of their treatment [3]. Radiotherapy employs ionizing radiation (IR), usually X-rays, to selectively eliminate targeted lesions [4]. The efficacy of radiotherapy relies on its physical mode of action, whereby IR interacts directly with atoms, resulting in molecular modifications of DNA, lipids, proteins, and other cellular components [5]. Furthermore, IR can induce indirect cellular effects by triggering the radiolysis of water molecules within cells, leading to the production of reactive oxygen species (ROS), such as hydroxyl radicals and hydrogen peroxide. These ROS can further damage nucleic acids, lipids, and proteins. Notably, DNA double-strand breaks induced by IR can trigger cell cycle arrest, senescence, and various forms of cell death, such as apoptosis, necrosis, mitotic catastrophe, autophagy [6], and ferroptosis [7], a regulated form of cell death characterized by lipid peroxidation [8].

Significant improvements have been made in the technical aspects of RT in recent years. The development of new irradiators and imaging systems has greatly enhanced the efficacy of RT while minimizing side effects. However, the radiation-induced damages to healthy tissues traversed by X-rays before to reach to the tumor mass. This limits the usable dose for patient treatment, making essential the development of new approaches to unleash the full therapeutic potential of RT, while preserving patient’s safety.

In 2011, NBTXR3 emerged as the first-in-class radioenhancer used in clinical practice for treating patients with locally advanced soft tissue sarcoma (NCT01433068) [9]. NBTXR3 also became the first radioenhancer to receive CE mark approval (Hensify®) after successfully completing a phase II/III clinical study (Act.in.Sarc study, NCT02379845) [10] and is currently being tested as single agent in several clinical trials including patients with head and neck squamous cell carcinoma (NCT01946867, NCT04862455) [11], pancreatic cancer (NCT04484909), lung cancer (NCT04505267), esophageal cancer (NCT04615013), or in combination with anti-PD1 (NCT05039632, NCT04862455, NCT03589339, or with chemotherapy (NCT04892173). NBTXR3 nanoparticles consist of a functionalized core of hafnium oxide, a high atomic number element, and were specifically designed to amplify the effects of RT within cells without additional side effects on healthy tissues [12, 13]. Leveraging this physical mechanism, radiation-activated NBTXR3 (NBTXR3 + RT) has demonstrated superior efficacy in destroying tumor cells and controlling tumor growth in numerous preclinical models and in human, surpassing the effects of RT alone, while maintaining a favorable safety profile [12, 14, 15]. Recent preclinical investigations have revealed that the benefits of NBTXR3 extend beyond mere radioenhancement and improved cancer cell destruction. Specifically, NBTXR3 has been reported to possess immunomodulatory properties through 1) improved induction of DNA damage, leading to better activation of the cGAS-STING pathway [16], 2) promotion of immunogenic cell death [17], 3) enhancement of immunopeptidome presentation [17], 4) generation of an antitumor immune response leading to the production of an abscopal effect mediated by CD8^+^ cytotoxic lymphocytes [18]. Notably, Hu et al. recently demonstrated in several studies using a two-tumor mouse model of anti-PD1-resistant lung cancer that the addition of NBTXR3 significantly improved the efficacy of various treatment regimens (RT + anti-PD1 and combinations with other check-point inhibitors) in terms of tumor growth, abscopal effect, and survival [19–22]. Recently, these authors also demonstrated the same benefits can be achieved when NBTXR3 was combined with proton therapy [23]. Moreover, all these studies (X-ray and proton) reported a robust activation of the antitumor immune response, restoration of the effectiveness of anti-PD1 therapy, and the induction of memory response in cured mice. Despite these promising outcomes, our understanding of the underlying biological events leading to these effects remains relatively limited.

In this article, we explored the early events triggered by the radioenhancing capacity of NBTXR3 and their potential role in explaining the superior efficacy of these nanoparticles in inducing cell death compared to RT alone.

## Materials and methods

### Cell culture and reagents

Human glioblastoma 42-MG-BA (#ACC-431) cell lines were purchased from DSMZ. HT1080 (#CCL-121, human fibrosarcoma), HCT116 (#CCL-247, human colorectal cancer), CT26.WT (#CRL-2638, mouse colorectal cancer), THP-1 (#TIB-202, human acute monocytic leukemia) and MDA-MB-231 (#HTB-26, human breast cancer) cell lines were purchased from the ATCC. For 42-MG-BA-ACTB-GFP cells establishment, 42-MG-BA parental cells were stably transfected by lipofectamine 2000 (#11,668, Invitrogen) with a pTagGFP2-actin vector (#FP194, Evrogen). The transfected populations were subjected to a selective pressure with G418 solution (#4,727,878,001, Roche). Stable transformants constitutively expressing the TagGFP2-actin fusion were used for in vitro assays described below. All cells were cultivated according to manufacturer’s recommendations and were controlled for mycoplasma by an independent laboratory (Clean Cells). NBTXR3 (Nanobiotix) is a sterile aqueous suspension of crystalized crystal hafnium oxide (HfO_2_) spherical nanoparticles with a size centered on 50 nm, determined by dynamic light scattering technique (Zetasizer NanoZS, Malvern Instruments Ltd), bearing a marked negative surface charge (–50 mV) in aqueous solution at pH 6–8 estimated by zeta potential analysis (Zetasizer NanoZS) [12].

### NBTXR3 labelling

Commercial dextran solution coupled with fluorochrome (#D3308 or #D1818, ThermoFisher Scientific) was diluted 1:500 (#D3308) or 1:100 (#D1818) in NBTXR3 suspension. The mix was incubated for 30 min at room temperature in the dark, then centrifuged. To remove unbound dextran, the pellet was resuspended in water. Nanoparticle suspension was sonicated for 15 min, then centrifuged. This washing step was repeated four additional times. After the last centrifugation, the final pellet (NBTXR3^RED^) was resuspended in water with the initial volume of NBTXR3 solution deposited, and sonicated for 30 min.

### Radiotherapy

All cells were irradiated using a 160 kV X-ray source (CP-160, Faxitron) at the dose and fraction number indicated for each study. For LysoTracker release assay, immuno-fluorescence assay and lipid peroxidation analysis, HCT116 and CT26.WT cells were irradiated using a 320 kV generator (X-RAD 320, Precision X-Ray).

### In vivo experiments

Six-week-old BALB/c female mice (Janvier Labs) were maintained under pathogen-free conditions in the animal facility at Brain Institute (Paris, France). For in vivo endocytose experiments, 3 × 10^5^ CT26.WT cells were subcutaneously injected into the right flank of mice. Length (L) and width (W) of tumors were measured with a digital caliper. Tumor volumes were calculated using the formula (LxW^2^/2). Once the tumor had grown (70 to 130 mm^3^), 4 to 5 mice were randomized into different groups. A volume of NBTXR3 suspension (or vehicle) corresponding to 25% of the baseline tumor volume was injected intratumorally. After 24 h, animals were euthanized by cervical dislocation, tumors were collected and dissociated with a tumor dissociation kit (#130–096-730, Miltenyi Biotec) using a GentleMacs Dissociator (Miltenyi Biotec). Fresh isolated cells were then used for further analysis described below. All animal experiments were carried out in compliance with French and European laws and regulations (European Directive 2010/63 EU). The local institutional animal ethics board and French Ministère de la Recherche approved all mouse experiments (permission numbers: 35858–2,022,102,115,492,949).

### Transmission electronic microscopy

For TEM analysis, 42-MG-BA, HT1080 or CT26.WT cells were seeded in 6-well plates. The next day, 400 µM NBTXR3 were added on cells for the duration indicated in each study. After this time, cells were pre-fixed with 1% glutaraldehyde (#EM-16220, Electron Microscopy Sciences) in 2.5% paraformaldehyde (#EM-15710, Electron microscopy sciences) for 1 h at room temperature. Samples were post-fixed during 1 h in 1% Osmium tetroxide (Electron Microscopy Sciences). Dehydration was performed for 10 min using graded series of ethanol (EtOH) in water 30%, 50%, 70%, 80%, 90% (× 2), 100% (× 3). Resin infiltration was performed by incubating 1 h in an agar low viscosity resin (Agar Scientific Ltd) and EtOH (1:2) mix, then 2 h in a resin and EtOH (1:1) mix followed by overnight incubation in pure resin. The resin was then changed, and the samples further incubated during 3 h prior to flat inclusion in gelatin capsules and overnight polymerization at 60 °C. Sections of 70 nm were obtained using an EM UC6 ultramicrotome (Leica), post-stained in 4% aqueous uranyl acetate and lead citrate and observed with a Tecnai12 transmission electron microscope equipped with a 4 K × 4 K Oneview camera (Gatan).

For Immuno-TEM analysis, 42-MG-BA cells were seeded in 75 cm^2^. The next day, 400 µM NBTXR3 or NBTXR3^RED^ were added to the flasks. The following day, cells were pre-fixed with 3.5% paraformaldehyde (#EM-15710, Electron Microscopy Sciences) for 2 h at room temperature and then overnight at 4 °C. Cells were rinsed three times in 0.2% glycine in PB buffer, covered in 2% gelatin in PB, scraped, centrifuged, and the pellets were taken up in 12% gelatin and kept on ice for 2 h. The pellets were cut into 1mm^3^ cubes and infused in 2.3 M sucrose overnight on a wheel at 4 °C. Four cubes per sample were mounted on nails and frozen in liquid nitrogen. Cryo-ultramicrotomy cuts were performed. Sections were incubated 15 min in 0.2% glycine in PB buffer. Blocking was performed in 0.1% CWFS gelatin for 30 min then the primary anti-LAMP-1 antibody (#555,798, BD Pharmingen) at a working dilution of 1/100 in 0.1% CWFS gelatin was added and incubated for 2 h at room temperature. The sections were then washed in 0.1% gelatin, six times for 5 min. Anti-mouse secondary antibody coupled to 10 nm gold beads (#810.022, Aurion) was diluted in 0.1% CWFS gelatin at a working dilution of 1/20, added to the sections and incubated for 2 h at RT. The sections were then washed in 0.1% gelatin, six times for 5 min, washed in PBS six times for 2 min, fixed in 1% glutaraldehyde in PBS for 5 min, washed in PBS three times for 2 min and then in H_2_O six times for 2 min. The contrast was performed in 0.4% uranyl acetate diluted in 2% methylcellulose first in a rapid bath and then for 5 min, then the sections were transferred into fresh drops of contrast. The contrast was absorbed on filter paper and the sections were dried. The sections were observed with a Tecnai12 transmission electron microscope equipped with a 4 K × 4 K Oneview camera (Gatan).

### Confocal microscopy

Cells were seeded in µ-Dish 35 mm petri dishes. The next day, fresh media containing NBTXR3^RED^ nanoparticles was added on cells for 24 h. Thirty minutes before acquisition, nuclei were counterstained with NucBlue Live ReadyProbes Reagent (#R37605, Invitrogen), actin was stained with 1 µM Cell Mask Green Actin (#A57243, Invitrogen) and lysosomes were stained with 75 nM Lysotracker Deep Red (#L12492, Invitrogen). Petri dishes were viewed under a confocal microscope (980 Airyscan FLIM) at the ImagoSeine platform at the Institut Jacques Monod. Images were processed with ImageJ software (v1.52a).

### LysoTracker and granularity kinetics

For LysoTracker signal and granularity kinetics analyses by flow cytometry, cells were seeded in 12-well plates at various density for each cell line. The next day, fresh media was applied on cells along with 400 µM NBTXR3 and then incubated at 37 °C and 5% CO_2_ during different times (30 min; 1 h; 3–4 h; 6 h; 16 h and 24 h). Cells were stained with 75 nM LysoTracker Green DND-26 (#L7526, Invitrogen) for 10 min in the dark at room temperature at the end of each time point. Finally, the Lysotracker signal and the Side Scatter (SSC-A) parameter were measured by flow cytometry (Accuri C6 Plus, BD Biosciences) to measure lysosomal staining evolution and the granularity of cells, respectively. To assess the effect of NBTXR3 concentration, 42-MG-BA cells were processed as just described but different concentration of NBTXR3 particles were added on cells (50; 100; 200; 400 and 800 µM).

For LysoTracker signal analyses by fluorescent microscopy, cells were seeded in µ-Dish 35 mm petri dishes. The next day, fresh media was applied along with 400 µM NBTXR3 and incubated during different times (30 min; 1 h; 3–4 h; 6 h; 16 h and 24 h). Cells were stained with 75 nM LysoTracker Green DND-26 (#L7526, Invitrogen) for 10 min in the dark at room temperature. Petri dishes were viewed under a fluorescent microscope (Zeiss Microscope AXIO Observer.D1). Images were processed with ImageJ Software (v1.52a).

### In vivo endocytosis analysis

Previously described fresh isolated cells from in vivo mice tumors were stained using a FITC anti-mouse CD45 antibody (1:100) (30-F11, BioLegend) for 45 min in the dark at room temperature. For lysosomes staining, cell suspension was incubated with 75 nM LysoTracker Deep Red (#L12492, Invitrogen) for 10 min in the dark at room temperature. Then, both the LysoTracker signal and the granularity (SSC-A parameter) of CD45^neg^ cells were analyzed by flow cytometry (AriaFusion, BD Biosciences). Finally, for each sample, CD45^neg^ were cell-sorted and processed for TEM analysis as previously described.

### Recapture assay

42-MG-BA parental cells seeded in T150 flask were treated with 400 µM of NBTXR3^RED^ particles. The next day, 42-MG-BA NBTXR3^RED^ positive cells were cell sorted by flow cytometry (AriaFusion, BD Biosciences) to discard remaining non-endocytosed NBTXR3^RED^ nanoparticles. Isolated 42-MG-BA NBTXR3^RED^ cells were then seeded in T25 flasks and irradiated or not, with 4 × 10 Gy. Right after the last fraction, 42-MG-BA-ACTB-GFP cells were added to irradiated 42-MG-BA NBTXR3^RED^ cells and incubated for 3 days. Then, the presence of NBTXR3^RED^ particles in 42-MG-BA-ACTB-GFP cells was assessed by flow cytometry (Accuri C6 Plus, BD Biosciences). Meanwhile, 42-MG-BA-ACTB-GFP and NBTXR3^RED^ positive cells were cell sorted (AriaFusion, BD Biosciences) and processed for TEM analysis as previously described.

For fluorescence microscopy analysis, the experiments were processed as previously described except that cells were seeded in µ-Dish 35 mm petri to allow microscopy acquisition. Images were acquired with a fluorescent microscope (Zeiss Microscope AXIO Observer.D1) and processed with ImageJ Software (v1.52a).

### Clonogenic assay

For clonogenic assays, cells were plated in triplicate into 6-well plates, then treated with 400 µM NBTXR3. After 16 h, a single dose of 4 Gy X-rays was delivered (or not) to cells, then the plates were placed in the incubator for 7–9 days. The colonies were fixed and stained with crystal violet (#V5265-500ML, Sigma). All colonies of ≥ 50 cells were then counted.

### LysoTracker release assay

Cells were seeded in 6-well plates. The next day, 400 µM NBTXR3 solution were added to the cell culture The following day, plates were irradiated or not with a 4 Gy single dose. After 1 h, fresh medium containing 75 nM LysoTracker Green DND-26 (#L7526, Invitrogen) was added to cells and incubated for 5 min. To assess the Lysosomal Membrane Permeabilization (LMP), LysoTracker signal was measured by flow cytometer (Cytoflex S, Beckman Counter; or Accuri C6 Plus, BD Biosciences) [24, 25].

### Immuno-fluorescence assay

For cathepsin release analysis, 42-MG-BA cells were seeded in µ-Dish 35 mm petri. The next day, media were replaced with fresh culture medium containing 400 µM of NBTXR3. The next day, cells were then irradiated with a single dose of 4 Gy. Cells were fixed by methanol 6 h after irradiation. Cathepsin D proteins were stained using a rabbit polyclonal antibody against cathepsin D (1:100) (ab75852, Abcam) and revealed with an Alexa Fluor 488-coupled anti-rabbit antibody (1:500) (ab150077, Abcam). Cells were viewed under a fluorescent microscope (Zeiss Microscope AXIO Observer.D1). Images were processed with ImageJ Software (v1.52a) according to the NIH recommendations (https://imagej.nih.gov/ij/docs/menus/analyze.html).

### Lipid peroxidation analysis

Cells were seeded in 6-well plates. The next day, 400 µM NBTXR3 solution were added to the cell culture. The next day, plates were irradiated or not with a 4 Gy single dose. At 24 h post-irradiation, fresh medium containing 10 µM of BODIPY®581/591 C11 dye (D3861, Invitrogen) was added to cells and incubated for 30 min. Lipid peroxidation level was measured by flow cytometer (Cytoflex S, Beckman Counter; or Accuri C6 Plus, BD Biosciences).

### RNAseq

For “OFF” status analysis, CT26.WT cells seeded in T75 flasks were incubated with 400 µM of NBTXR3. The next day, cells were collected, washed and dry cell pellets were cryopreserved for bulk RNA sequencing. For “ON” status, CT26.WT cells seeded in T75 flasks were incubated with 400 µM of NBTXR3. The next day, cells were irradiated or not, with a single dose of 4 Gy. After 24 h, cells were collected, washed and dry cell pellets were cryopreserved for bulk RNA sequencing. The bulk RNA (polyA) sequencing was performed by LC Sciences (Houston). Briefly, Poly(A) RNA sequencing library was prepared following Illumina’s TruSeq-stranded-mRNA sample preparation protocol. RNA integrity was checked with Agilent Technologies 2100 Bioanalyzer. Poly(A) tail-containing mRNAs were purified using oligo-(dT) magnetic beads with two rounds of purification. After purification, poly(A) RNA was fragmented using divalent cation buffer in elevated temperature. The DNA library construction is shown in the following workflow. Quality control analysis and quantification of the sequencing library were performed using Agilent Technologies 2100 Bioanalyzer High Sensitivity DNA Chip. Paired-ended sequencing was performed on Illumina’s NovaSeq 6000 sequencing system.

### Statistical analysis

In vitro studies have been independently repeated at least three times. Results are expressed as mean ± SEM. Normality distribution of values was assessed by Shapiro–Wilk normality test. Experiments with normal distribution were analyzed by One-way ANOVA. Experiments with non-normal distribution were analyzed by Friedman test. A *p* value < 0.05 was considered statistically significant. The software GraphPad Prism 9® v.9.4.1 (681) was used for graph plotting and biostatistics.

For RNAseq analysis, unless stated otherwise, data analyses were conducted using various R v4.2.1 system software packages, including those from Bioconductor (Bioc 3.16 Released), or custom R code. Appropriate R packages were specified when applicable. For gene filtering, we excluded genes with less than 10 counts in any one sample out of the six we had. Then, read counts were normalized by the variance stabilizing transformation vst function from DESeq2 (v1.38.3) R package [26]. In purpose to explore the gene expression level involve into a specific functions (such as lysosomes, oxidative stress,…), the Gene Ontology (GO) knowledgebase dated July 1, 2022 was collected from GO.db (v3.16.0) R package. The gene expression levels were represented using a heatmap with Z-scores calculated on a gene-by-gene basis. The Z-scores were computed by subtracting the mean and dividing by the standard deviation, and the heatmap was generated using the Heatmap function from the ComplexHeatmap (v2.14.0) R package. To identify genes differentially expressed between groups and estimate the fold change, we performed a linear model and applied empirical Bayes smoothing to the standard errors (lmFit and eBayes function from limma v3.54.2 R package [27, 28] on vst transformation gene expression dataset. Resulting p values were adjusted for multiple hypothesis testing with Benjamini–Hochberg procedure [29].

## Results

### Cancer cells quickly internalize NBTXR3 nanoparticles through macropinocytosis and the clathrin-dependent pathway

**Fig. 1:**
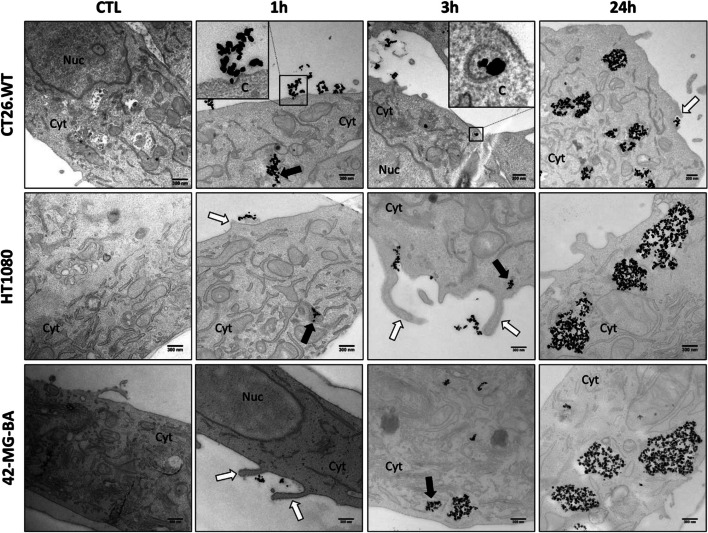
NBTXR3 nanoparticles are internalized by different endocytosis pathways. Transmission electronic microscopy (TEM) representative images of NBTXR3 nanoparticles uptake by macropinocytosis and clathrin-mediated endocytosis in CT26.WT (upper panel), HT1080 (middle panel) and 42-MG-BA (lower panel) at 1 h, 3 h and 24 h after addition of 400 µM NBTXR3. The membrane invagination and lamellipodia observed indicate membrane perturbations typical of macropinocytosis. White arrow: membrane ruffles enclosing a small number of NBTXR3 nanoparticles; black arrow: internalized NBTXR3 nanoparticles cluster in early endosomes. Scale bar, 300 nm. Abbreviations: C, Clathrin; CTL, control cells (no NBTXR3); Cyt, cytoplasm; Nuc, nucleus

First, we aimed to determine the mechanisms of endocytosis involved in the internalization of NBTXR3 nanoparticles by cancer cells. For this purpose, we conducted a series of transmission electron microscopy (TEM) analyses at 1 h, 3 h, and 24 h post-treatment with NBTXR3 on human cell lines HT1080, 42-MG-BA, and murine cell line CT26.WT (Fig. 1, Supplemental Fig. S1). Examination of resulting images allowed us to identify two endocytic pathways: macropinocytosis and clathrin-dependent endocytosis. Macropinocytosis was observed in all three tested cell lines, as evidenced by membrane invaginations and characteristic lamellipodia associated with this endocytic pathway [30]. Particularly, at 3 h post-treatment in the HT1080 cell line, contiguous NBTXR3 nanoparticles were observed at the cell membrane, with a lamellipodium closing around them.

In the CT26.WT cell line, thickening of the plasma membrane invaginated towards the interior of the cell (seen at 1 h), along with the presence of an endosome displaying a membrane thicker than the plasma membrane (seen at 3 h), indicated features characteristic of clathrin-dependent endocytosis. However, these observations were only sporadically observed only in this cell line, suggesting that this pathway may not be predominantly used for internalizing NBTXR3 nanoparticles compared to macropinocytosis.

These analyses also demonstrated that the endocytosis of NBTXR3 nanoparticles is a relatively fast process. Indeed, as early as 1 h post addition of NBTXR3 on cells, nanoparticles were detectable in the cytoplasm of all three cell lines (Fig. 1, Supplemental Fig. S1). The majority of nanoparticles appeared grouped in the cytoplasm, forming clusters encompassing up to a few tens of nanoparticles. This specific configuration of nanoparticles in the cytoplasm suggests the simultaneous internalization of multiple nanoparticles, consistent with macropinocytosis. The presence of large clusters (containing several hundreds of nanoparticles) was predominantly observed at 24 h in all three cell lines, suggesting that the smaller clusters observed at 1 h and 3 h fused over time to form these larger clusters.

These clusters are enclosed by membranes, indicating their presence within cytoplasmic vesicles rather than being freely dispersed in the cytoplasm. The contents of these vesicles containing NBTXR3 displayed a generally homogeneous appearance across the cell lines, despite a few multivesicular bodies being observed (Supplemental Fig. S1). Finally, the distribution of NBTXR3 clusters in the cytoplasm appears to be relatively random. However, it is worth noting that some clusters are located in very close proximity to the nucleus at 24 h (Supplemental Fig. S1).

### Cancer cells have different NBTXR3 endocytosis capacities and rates

**Fig. 2:**
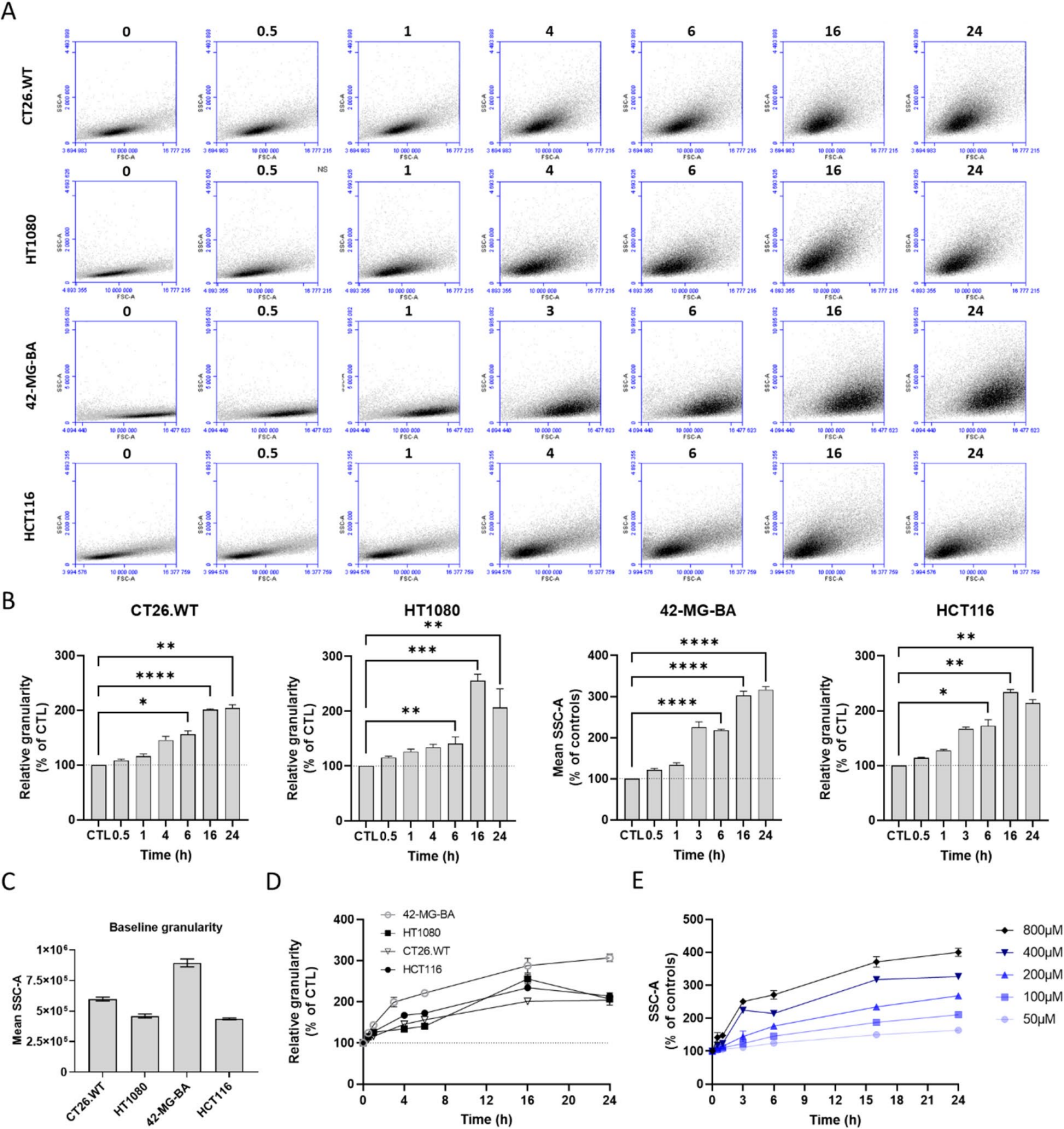
Endocytosis of NBTXR3 nanoparticles by cancer cells increases constantly over time. Following addition of 400 µM NBTXR3, the evolution of the cellular granularity at the indicated time-points was carried out by flow cytometry for the CT26.WT, HT1080, 42-MG-BA, and HCT116 cells. **A** Representative flow cytometry analysis of the evolution of the cellular granularity over time. **B** Kinetic of cellular granularity profile evolution for the CT26.WT (*n* = 4), HT1080 (*n* = 5), 42-MG-BA (*n* = 8), and HCT116 (*n* = 3) cells. Data of independent experiments are represented as the relative granularity to CTL ± SEM. Statistical test: One-way ANOVA. *, *p* < 0.05, **, *p* < 0.01, ***, *p* < 0.001, ****, *p* < 0.0001. **C** Baseline granularity of the tested cell lines. Data of independent experiments are represented as the mean granularity (SSC-A) ± SEM. **D** Comparison of granularity profile evolution for tested cell lines. Data of independent experiments are represented as the relative granularity to CTL ± SEM. **E** Evolution of the cellular granularity profile for 42-MG-BA cells (*n* = 4) according to NBTXR3 concentrations

We then wanted to determine whether the endocytosis rate of NBTXR3 nanoparticles was comparable among the different tested cell lines and whether it exhibited a continuous or saturable phenomenon. To address this, we took advantage of the increased cell granularity resulting from the presence of nanoparticles in the cytoplasm after endocytosis, which can be measured by flow cytometry (Fig. 2) [31]. Indeed, the more NBTXR3 nanoparticles a cell internalized, the higher its granularity. Based on this principle, we performed an endocytosis kinetic assay following NBTXR3 addition using CT26.WT, HT1080, 42-MG-BA, and HCT116 cells. Figure 2A shows a representative profile of the time-dependent changes in granularity for each of the four cell lines studied. This analysis revealed a clear increase in cell granularity over time, regardless of the cell line considered, which is in good agreement with the previous TEM analyses (Fig. 1). Figure 2B illustrates the time-dependent average cellular granularity measured across multiple independent experiments (n ≥ 3). We observed a continuous increase in granularity for all cell lines till 16 h post-treatment. Similar profiles were obtained for MDA-MB-231 and THP-1 cells (Supplemental Fig. S2). At this time point, the baseline granularity of CT26.WT, HT1080, 42-MG-BA, and HCT116 cells increased by 101 ± 3.1%, 155 ± 26.4%, 203 ± 28.8%, and 134 ± 8.0%, respectively. At 24 h post-treatment, the granularity of HCT116 and HT1080 cells started to decrease, CT26.WT cells reached a plateau, while the granularity of 42-MG-BA cells continued to increase. The decrease in granularity for HCT116 and HT1080 cell lines could be due to cell division, which redistributes the NBTXR3 nanoparticles among sister cells, naturally leading to a decrease in the granularity of each new individual cell [32, 33]. For the other cell lines, the disparity in profiles observed at 24 h post-treatment can be attributed to longer cell division times. For example, HCT116 and HT1080 cells have a cell doubling time of 22 and 26 h [34, 35], respectively, whereas 42-MG-BA cells take twice as long to divide [36]. Note that at base line, these cell lines displayed various levels of granularity, which could be correlated with cell division time (Fig. 2C). For example, the mean granularity of 42-MG-BA cells at baseline (894,912 ± 32,503) is more than 2 times superior to the one of HCT116 cells (435,971 ± 13,989).

Figure 2D shows the relative variable endocytosis rates among the cell lines tested, compared to untreated cells. For time points ≤ 6 h, the endocytosis rate and capacity of 42-MG-BA cells are markedly higher than the other three cell lines, which exhibit similar endocytosis profiles overall. After 6 h, there is a decrease in the slope of granularity for all cell lines, although the rate of NBTXR3 endocytosis remains relatively high for the 42-MG-BA cell line. This indicates a slowing down of nanoparticle endocytosis velocity, which could be attributed to a reduction in endocytic capacity. However, this slowdown could be more likely linked to a decrease in the proximity of available nanoparticles for endocytosis. This possibility is supported by results presented in Fig. 2E, showing that the increase in granularity of 42-MG-BA cells was directly related to the concentration of NBTXR3. For example, doubling the concentration of NBTXR3 delivered to the cells (from 400 µM to 800 µM) did not reveal any signs of saturation of the endocytosis system of these cells.

### NBTXR3-containing endosomes are fused with lysosomes

**Fig. 3:**
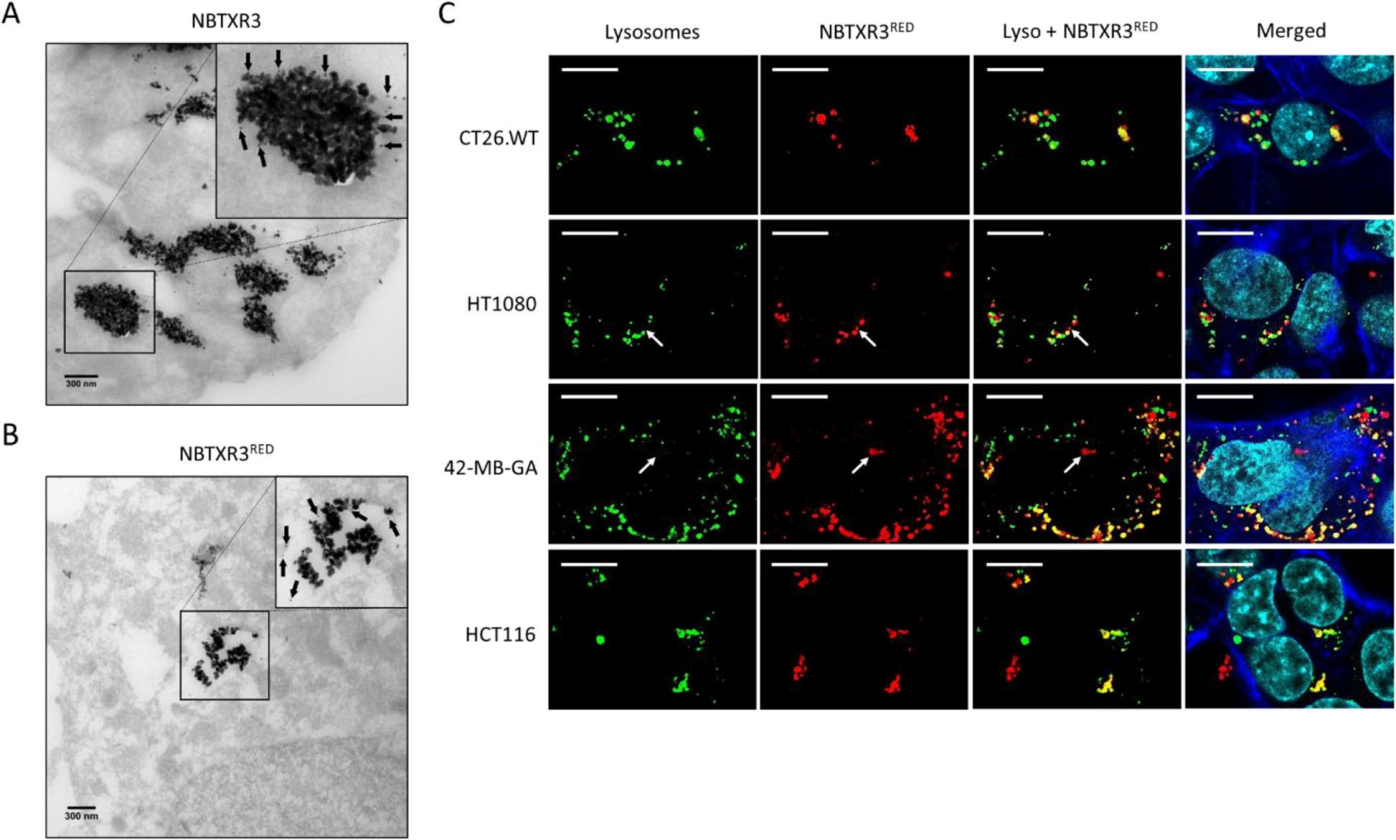
Endosomes containing NBTXR3 clusters are fused with lysosomes. Immuno-TEM images obtained 24 h after treatment of 42-MG-BA cells, showing that **A** NBTXR3 and **B** NBTXR3^RED^ clusters colocalize with LAMP-1, a specific marker for lysosomes. Black arrows indicate the location of AuNPs allowing identification of LAMP-1, surrounding the clusters of NBTXR3 and NBTXR3^RED^. Scale bar, 300 nm. **C** Representative confocal microscopy images taken 24 h after treatment of CT26.WT, HT1080, 42-MG-BA and HCT116 cells with NBTXR3.^RED^  ., LysoTracker-stained lysosomes  ;, NBTXR3 nanoparticle labelled with utilized Dextran Tetramethylrhodamine (NBTXR3^RED^);  , actin filaments;  , nucleus. Scale bar, 10µm

The findings presented in Fig. 1 demonstrate that NBTXR3 nanoparticles were internalized by macropinocytosis and, to a lesser extent, by clathrin-dependent endocytosis in various cell types. Typically, the intracellular trafficking of endosomes formed through macropinocytosis and clathrin-dependent endocytosis pathways lead to their fusion with lysosomes. To verify that NBTXR3-containing endosomes are targeted to lysosomes, we conducted an immuno-TEM analysis on 42-MG-BA cells 24 h after the addition of the nanoparticles. This analysis involved the use of an anti-LAMP-1 antibody, which serves as a specific marker for lysosomes [37]. Figure 3A depicts the localization of LAMP-1 (gold nanoparticles (AuNP)) surrounding NBTXR3 clusters, providing evidence for the presence of NBTXR3 nanoparticles within lysosomes.

Currently, TEM analysis is the only technique available for direct visualizing of NBTXR3 nanoparticles inside cells. However, while the previous findings confirm the presence of NBTXR3 in lysosomes, TEM does not enable a straightforward and comprehensive assessment of the proportion of NBTXR3 nanoparticles present in lysosomes. To overcome this limitation, we utilized NBTXR3 labelled with dextran tetramethylrhodamine (NBTXR3^RED^) as an indirect method to visualize NBTXR3 nanoparticles using fluorescence microscopy (Supplemental Fig. S3A and S3B). Immuno-TEM analysis using anti-LAMP-1 antibodies demonstrated that NBTXR3^RED^ nanoparticles were still present within lysosomes, indicating that the labeling process did not interfere with the trafficking pattern previously observed for NBTXR3 (Fig. 3B). Confocal microscopy experiments demonstrated that, 24 h after the addition of NBTXR3^RED^ to 42-MG-BA, HCT116, HT1080, and CT26.WT cells, a vast majority of NBTXR3^RED^ clusters showed colocalization with lysosomes (Fig. 3C). A small number of NBTXR3^RED^ endosomes did not exhibit colocalization with lysosomes (Fig. 3C, white arrows). This could be related to 1) escape of NBTXR3^RED^ from endosomes/lysosomes, 2) endocytosis of NBTXR3^RED^ through a pathway that bypasses endosomes and targets different compartments (e.g., lipid-raft endocytosis), but more likely 3) recent endocytosis of NBTXR3^RED^ where fusion with a lysosome has not yet occurred. Furthermore, these images confirm that NBTXR3 clusters were generally randomly distributed in the cell cytoplasm, with some of them being in very close proximity to the nucleus but never found inside.

### NBTXR3 increases lysosomal content

**Fig. 4:**
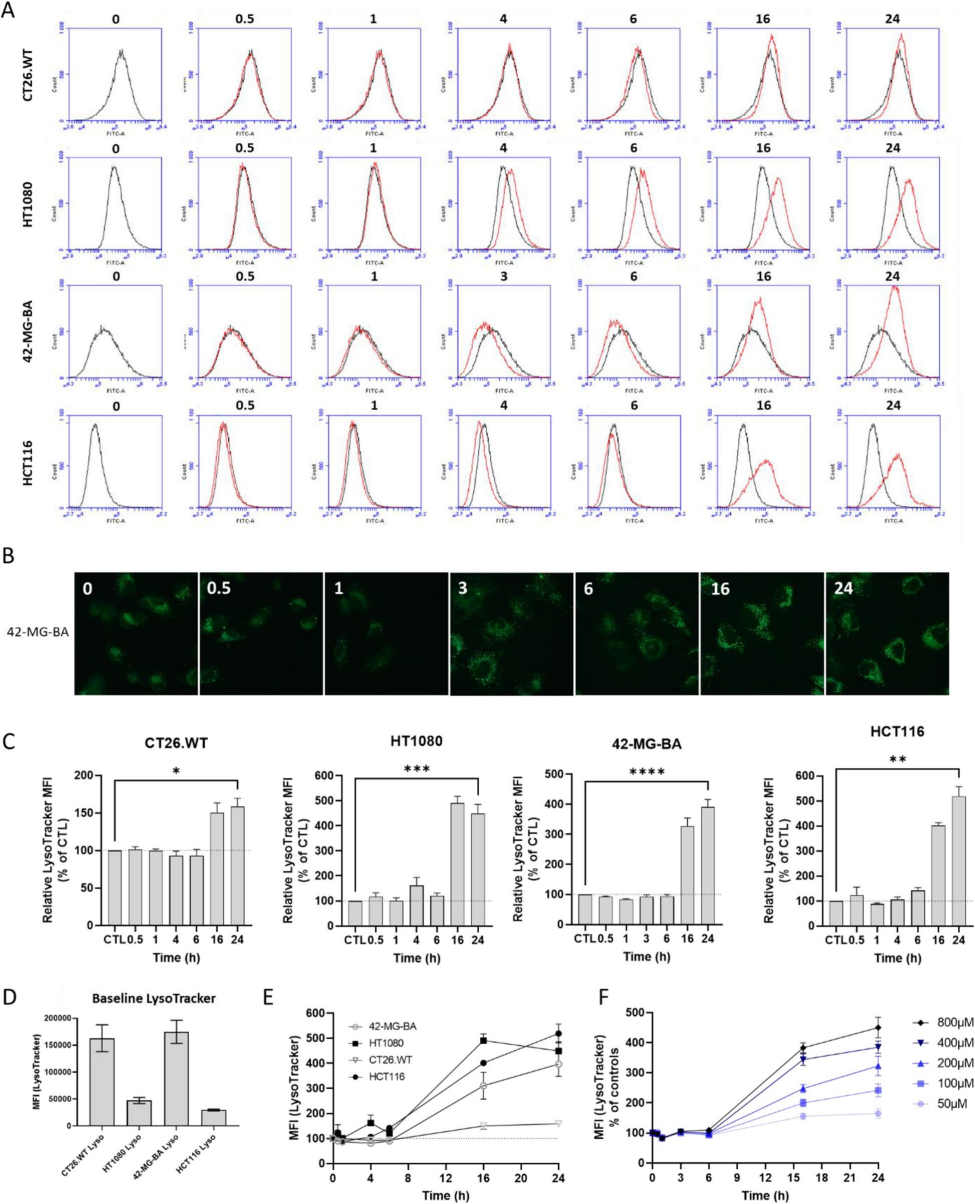
Endocytosis of NBTXR3 nanoparticles by cancer cells increases LysoTracker signal over the time. Following addition of 400 µM NBTXR3, the evolution LysoTracker signal at the indicated time-points was carried out by flow cytometry for the CT26.WT, HT1080, 42-MG-BA, and HCT116 cells. Representative evolution of LysoTracker signal over time analyzed by **A** flow cytometry and **B** fluorescent microscopy. **C** Kinetic of modification of LysoTracker profile for the CT26.WT (*n* = 4), HT1080 (*n* = 5), 42-MG-BA (*n* = 8), and HCT116 (*n* = 3) cells. Data of independent experiments are represented as the relative LysoTracker MFI to CTL ± SEM. Statistical test: One-way ANOVA or Friedman test. *, *p* < 0.05; **, *p* < 0.01; ***, *p* < 0.001, *p* < 0.0001; ****. **D** Baseline LysoTracker signal of the tested cell lines. Data of independent experiments are represented as the MFI ± SEM. **E** Comparison of MFI LysoTracker profile evolution for tested cell lines. Data of independent experiments are represented as the relative MFI to CTL ± SEM. **F** Evolution of the MFI LysoTracker profile for 42-MG-BA cells (*n* = 4) according to NBTXR3 concentrations

Next, we investigated the potential impact of NBTXR3 endocytosis on the lysosomal content of cells. To achieve this goal, we stained lysosomes with LysoTracker and monitored the fluorescence changes over time using flow cytometry (Fig. 4). Figure 4A shows a representative profile of the LysoTracker fluorescence signal over time measured by flow cytometry for each of the four cell lines studied. Figure 4B shows representative fluorescent microscopy pictures obtained at the same timepoints, for 42-MG-BA cells (see Supplemental Fig. S4 for CT26.WT, HT1080 and HCT116 cells). In Fig. 4C, the relative evolution of lysosomal mean fluorescence intensity (MFI) over time is depicted based on multiple independent experiments (n ≥ 3). These figures reveal that at the early timepoints following the addition of NBTXR3 (≤ 6 h), the MFI tends to slightly decrease for all cell lines except HT1080. Similar profiles were obtained for MDA-MB-231 and THP-1 cells (Supplemental Fig. S5). The reasons why this phenomenon was not seen in HT1080 cells remain to be elucidated, but for all other cell lines, the slight decrease in LysoTracker signal could be explained by the fusion of lysosomes with endocytic vesicles containing NBTXR3. The fusion of lysosomal content with these vesicles, which have a higher pH compared to lysosomes, could impact the LysoTracker signal, which stain only acidic compartments (lysosomes). From 16 h, regardless of the cell line considered, a significant increase in MFI can be observed. The magnitude of this fluorescence gain varies depending on the cell line. For instance, the signal increase is less pronounced in CT26.WT cells. This must be elucidated, but cannot be attributed to the high baseline levels of LysoTracker signal in these cells, which is 3 to 4 times higher compared to HT1080 and HCT116 cells (Fig. 4D). Indeed, 42-MG-BA cells have a LysoTracker profile like HT1080 and HCT116 cells after treatment with NBTXR3, despite a LysoTracker signal at baseline equivalent to the one measured for CT26.WT cells.

Figure 4E indicates that the increase in LysoTracker signal profile over time is relatively similar for 42-MG-BA, HT1080 and HCT116, compared to CT26.WT cells. In the case of HT1080 cells, the observed loss of LysoTracker signal at 24 h aligns with the loss in granularity, most likely due to cell division. It is also interesting to note that the intensity of the LysoTracker signal increased as a function of the concentration of NBTXR3 applied to the 42-MG-BA cells (Fig. 4F). The more this concentration increases, the more the MFI increases for the times 16 and 24 h post-treatment. This is in good agreement with previously observed granularity results for this cell line at these time points (Fig. 2E).

**Fig. 5:**
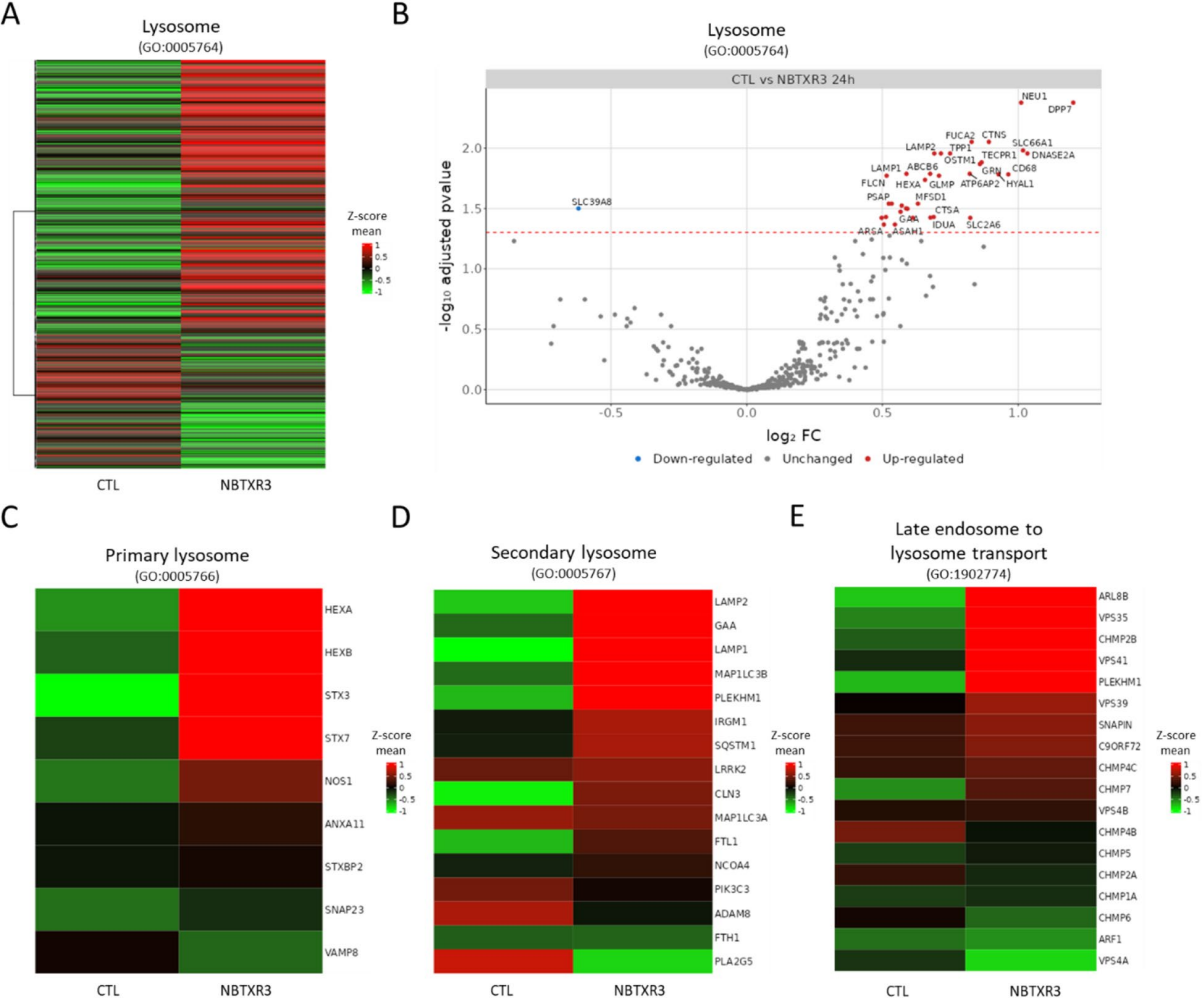
Endocytosis of NBTXR3 nanoparticles induces upregulation of genes linked to lysosomes. RNAseq analysis of CT26.WT cells treated for 24 h with NBTXR3 (*n* = 3). **A** Heatmap of lysosome-associated genes (GO:0005764). **B** Volcano plot of lysosome-associated genes (GO:0005764) showing significantly differentially expressed genes (*p* < 0.05) of NBTXR3-treated (NBTXR3) vs. untreated (CTL) CT26.WT cells. Heatmap of expression of **C** primary lysosome (GO:0005766) **D** secondary lysosome (GO:0005767) and **E** late endosome to lysosome transport (GO:1,902,774). For all presented heatmaps, each column stands for the mean value of three independent experiments. Expression values are shown as the Z-score mean of the log_2_ transformed normalized counts. Red and green are assigned to higher and lower expression, respectively, according to the color key in the right

We then analyzed the transcriptome of CT26.WT cells exposed for 24 h to NBTXR3 (Fig. 5). Our data revealed a global increase in the transcription of genes associated with lysosomes (Fig. 5A and B, Supplemental Table 1). Among them, 34 genes had their transcription significantly increased compared to control (Fig. 5B). These genes encode proteins associated with the lysosomal membrane (e.g., *Cd68*, *Lamp1*, *Lamp2*), acidification of lysosomes (e.g., *Abcb6*, *Atp6ap2*, *Atp6v0d1*, *Mcoln1*), lysosomal acid hydrolases including proteases (e.g., *Ctsa*, *Ctsd*, *Dpp7*, *Tpp1*), glycosidases (e.g., *Dpp7, Fuca2*, *Gaa*, *Gba*, *Hyal1*, *Idua*, *Neu1*, *Tpp1*), sulfatase (e.g., *Arsa*), lipases (e.g., *Asah1*, *Hexa*), DNAse (e.g., *Dnase2a*), but also genes related to the biogenesis of lysosomes (e.g., *Glmp*, *Ostm1)* and lysosomal transport of various elements (*Ctns*, *Slc2a6*). The activation of these genes indicates that cells have adapted their metabolism following nanoparticle endocytosis to produce elements participating in lysosome functions and biogenesis. This hypothesis is supported by the overall increase in the expression of genes involved in both primary lysosomes (Fig. 5C) and secondary lysosomes (Fig. 5D). Primary lysosomes are newly formed small vesicles that originate from the Golgi apparatus and typically contain inactive digestive enzymes, while secondary lysosomes are formed when primary lysosomes fuse with endosomes, such as phagosomes or pinosomes. These results are in good agreement with the previously observed increase in LysoTracker signal (Fig. 4). It is also interesting to note the increase in the expression of *Arl8b* and *Vps35*, two genes involved in endosomes/lysosomes fusion (Fig. 5E), which is consistent with our above-mentioned immuno-fluorescence observations (Fig. 3C). Only *Scl39a8* gene involved in Zinc transport has its transcription significantly reduced (Supplemental Table 1). The biological significance of this downregulation has to be investigated.

### NBTXR3 increases granularity and LysoTracker signal of cancer cells in tumor

**Fig. 6:**
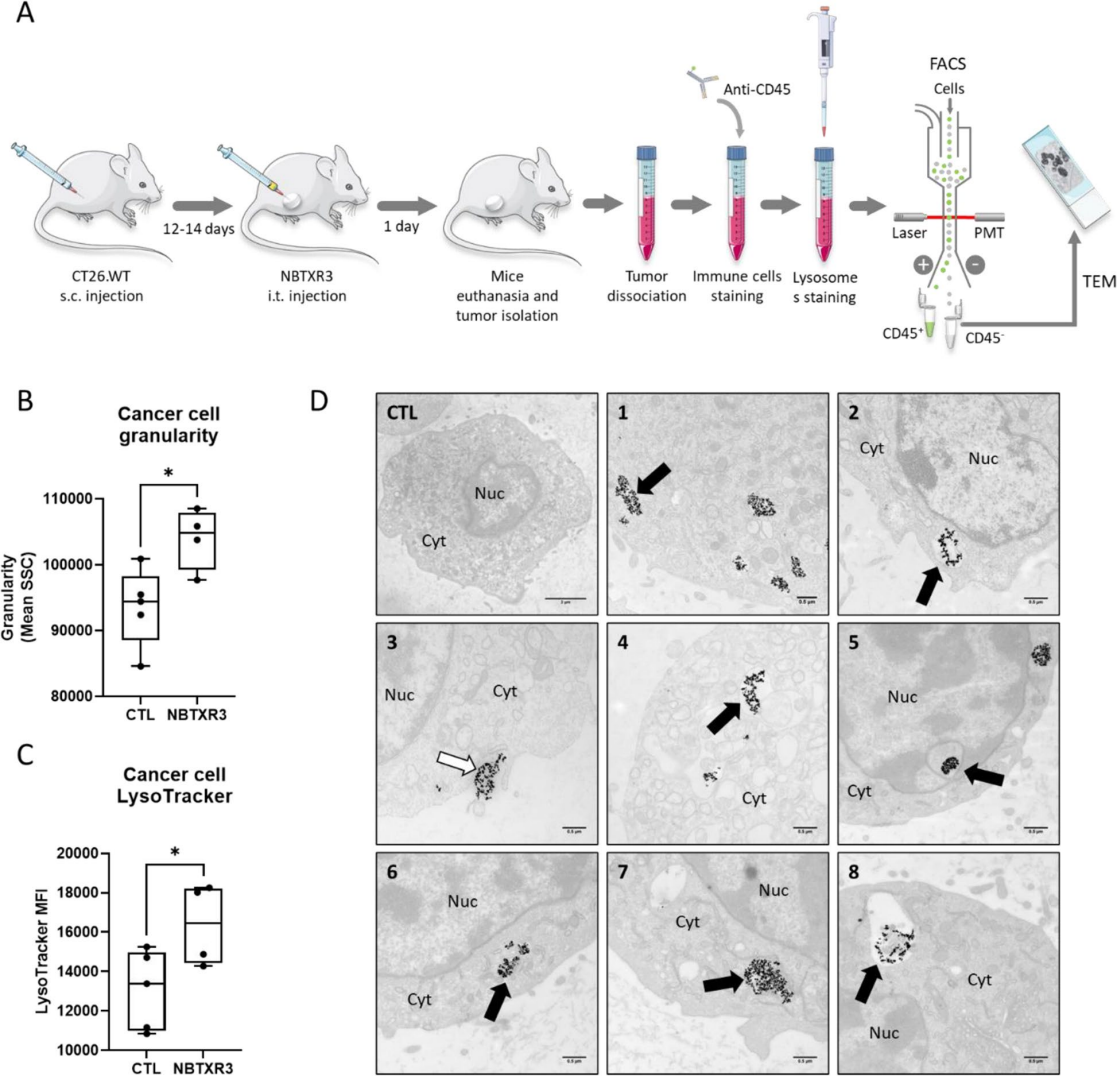
Intratumoral injection of NBTXR3 lead to significant increase of both granularity and LysoTracker signal, and endocytosis by cancer cells. **A** Schematic representation of the experiment workflow. Box and whisker plots representation of cancer cells **B** granularity and **C** LysoTracker signal measured 24 h after intratumoral injection of NBTXR3 or 5% Glc (vehicle, CTL). Presented data were obtained from 4–5 mice per group (each dot represents one value) from two independent experiments. Statistical test: Unpaired t-test. *, *p* < 0.05. **D** TEM representative images of NBTXR3 nanoparticles uptake by CT26.WT cells 24 h after intratumoral injection of NBTXR3 from five mice (CTL, mouse #1; NBTXR3-treated mice: mouse #2, pictures 1–2; mouse #3, picture 3; mouse #4, picture 4; mouse #5, pictures 6–8). White arrow: membrane ruffles enclosing a small NBTXR3 nanoparticle aggregate; black arrow: internalized NBTXR3 nanoparticles cluster. Scale bars: CTL, 2µm; 1–8, 0.5µm. Abbreviations: CTL, control cells (no NBTXR3); Cyt, cytoplasm; Nuc, nucleus

In patients, NBTXR3 nanoparticles are delivered via a single intratumoral injection before radiotherapy. Within a tumor, cancer cells evolve in a complex 3D environment (hypoxia, nutrient deficiency, immune cells, intratumoral pressure, etc.) that is very different from the controlled 2D conditions of in vitro studies. To confirm that our in vitro findings (i.e., increased granularity and Lysotracker signal) occurred in the same way in a tumor context, we performed intratumoral injections of NBTXR3 nanoparticles in immunocompetent mice bearing a single subcutaneous CT26.WT tumors (Fig. 6). The next day, following the steps outlined in Fig. 6A, we isolated cancer cells. This analysis revealed a significant increase in granularity (Fig. 6B) and LysoTracker MFI (Fig. 6C) in these cancer cells, like what was previously observed in vitro (Figs. 2 and 4). To confirm that the increased granularity and LysoTracker MFI in these cells were indeed associated with the presence of NBTXR3 nanoparticles in their cytoplasm, we isolated by FACS the cancer cells with higher granularity compared to cells from control tumors for TEM analysis (Fig. 6D). TEM images obtained from these isolated cells clearly showed the presence of NBTXR3 clusters in the cytoplasm, similar to those previously observed in in vitro analyses (Fig. 1). These findings strongly suggest that the in vivo mechanisms of endocytosis and intracellular trafficking on NBTXR3 are comparable to those we reported in vitro. This also suggests that other in vitro observations (lysosomal content increase) also occur within the tumor.

### NBTXR3 released after cell death can be re-endocytosed

**Fig. 7:**
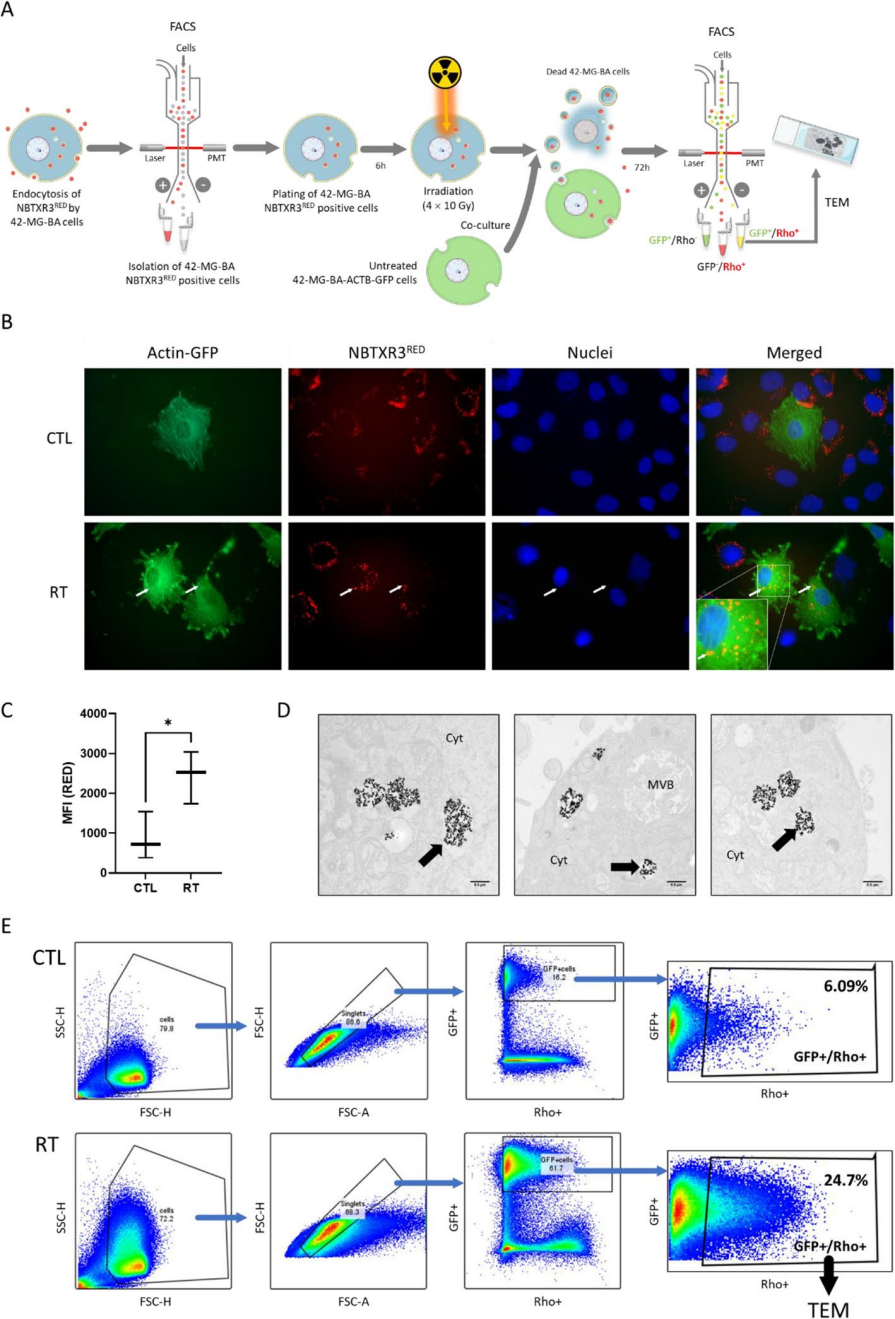
NBTXR3 nanoparticles released from cancer cells killed by RT can be again endocytosed by live cancer cells. Analysis of recaptures NBTXR3^RED^ released from 42-MG-BA dead cells. **A** Schematic representation of the experiment workflow. **B** Fluorescent microscopy representative images of NBTXR3^RED^ nanoparticles recapture experiment 48h after co-culture of untreated (CTL, upper panel) or RT-treated (RT, lower panel) conditions. White arrows indicate the presence of NBTXR3^RED^ nanoparticles in the cytoplasm of 42-MG-BA-ACTB-GFP cells. **C** Box and whisker plots representation of flow cytometry analysis of NBTXR3^RED^ MFI in 42-MG-BA-ACTB-GFP cells according to the tested condition (CTL, untreated; RT, radiotherapy-treated), from three independent experiments. Statistical test: Paired t-test. *, *p* < 0.05. **D** Representative TEM images of NBTXR3^RED^ nanoparticles uptake by 42-MG-BA-ACTB-GFP cells 48h after co-culture with dead 42-MG-BA NBTXR3^RED^ cells. Black arrow: internalized NBTXR3^RED^ nanoparticles cluster. Scale bar: 0.5µm. Abbreviations: Cyt, cytoplasm; MVB, multivesicular body. **E** FACS gating strategy employed to isolate NBTXR3^RED^-containing 42-MG-BA-ACTB-GFP cells from RT condition for TEM analysis

The ability of NBTXR3 activated by RT to more effectively destroy cancer cells and better control tumors than RT alone has been demonstrated in numerous in vitro and in vivo models [12, 14, 15, 19–22] as well as in humans [9, 38]. It has also been shown that once injected into the tumor, there is a high retention of NBTXR3 nanoparticles that do not diffuse out of it [12, 15, 18]. Even several months after complete tumor destruction in patients, the nanoparticles are still present at the injection site [38]. This raises the question of the fate of the nanoparticles after the destruction of cancer cells. We hypothesized that after the destruction of the tumor cell, the released nanoparticles could be recaptured by other viable tumor cells, despite a biologically complex environment surrounding these released nanoparticles (e.g., cellular debris, nanoparticles encapsulation, etc.), which is very different from the initial injection context. To test this hypothesis, we followed the steps described in Fig. 7A. Thanks to the sequential utilization of 42-MG-BA cells, 42-MG-BA-ACTB-GFP cells and NBTXR3^RED^ nanoparticles and performing microscopy analysis (Fig. 7B) and flow cytometry (Fig. 7C and E), we were able to confirm this hypothesis. Indeed, in Fig. 7B, it can be noticed that no NBTXR3^RED^ nanoparticles were detected in 42-MG-BA-ACTB-GFP cells co-cultured with NBTXR3^RED^-pretreated unirradiated 42-MG-BA cells (CTL condition). In stark contrast, the presence of NBTXR3^RED^ can be observed in the 42-MG-BA-ACTB-GFP cells when co-cultured with NBTXR3^RED^-pretreated irradiated 42-MG-BA cells (RT condition), showing that NBTXR3^RED^ released from dead cells can be recaptured by other cancer cells. To assess this phenomenon on a larger number of cells, we analyzed the evolution of MFI induced by the presence of NBTXR3^RED^ in the 42-MG-BA-ACTB-GFP cells using flow cytometry (Fig. 7C and E). It can be seen that in the RT condition, the NBTXR3^RED^ signal significantly increases in 42-MG-BA-ACTB-GFP compared to the control condition. Interestingly, TEM analysis (Fig. 7D, Supplemental Fig. S6) shows that within 42-MG-BA-ACTB-GFP cells that have recaptured the nanoparticles, they reform clusters as previously observed in in vitro studies (Fig. 1) and in vivo studies (Fig. 6), suggesting that the previously described biological processes may occur again in.

### NBTXR3 activated by RT triggers lysosomal membrane permeabilization cell and ferroptosis

**Fig. 8:**
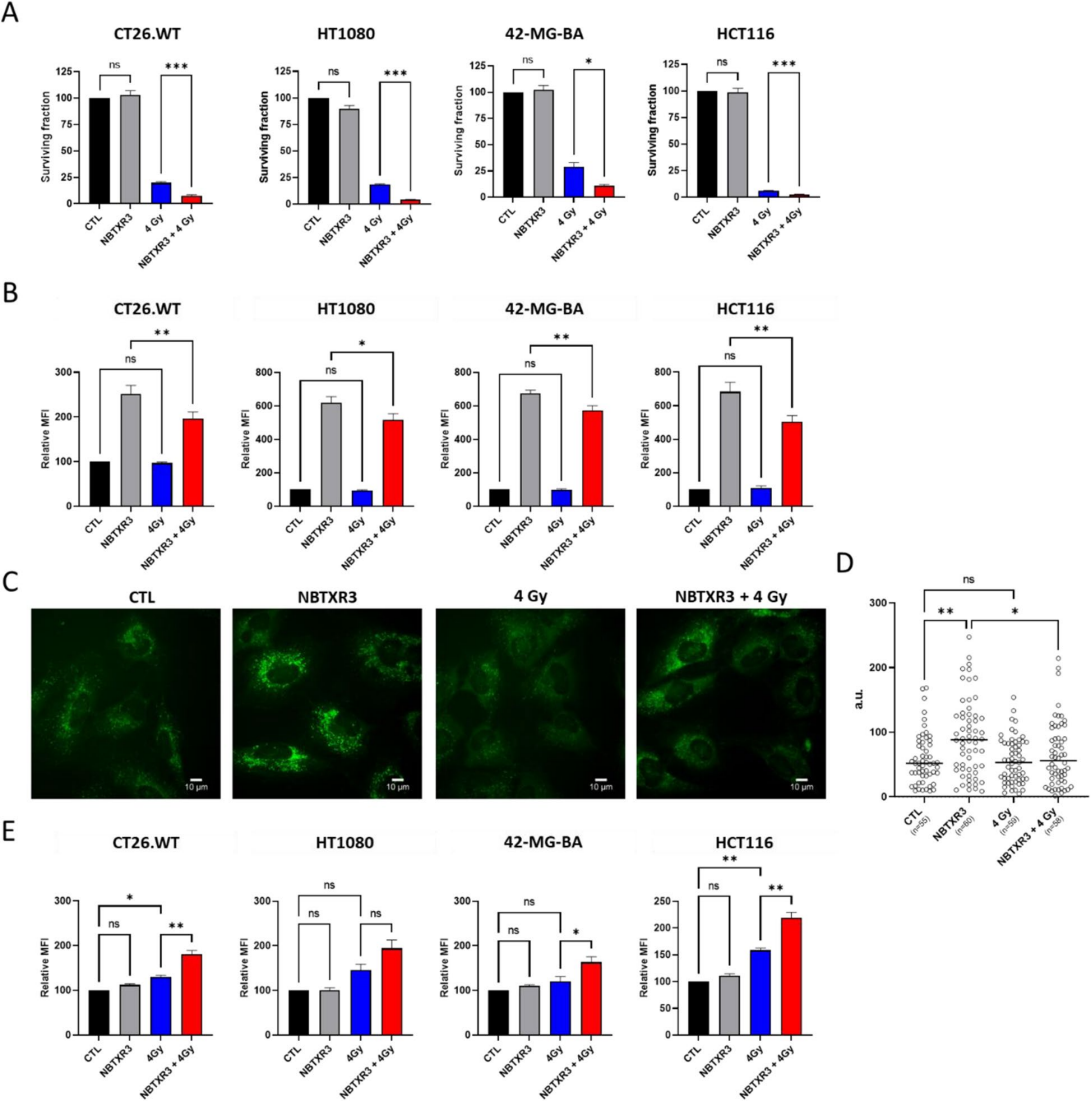
NBTXR3 + RT promotes ferroptosis cell death through lysosomal membrane permeabilization and lipid peroxidation enhancement. **A** Clonogenic assay on CT26.WT (*n* = 5), HT1080 (*n* = 4), 42-MG-BA (*n* = 3) and HCT116 (*n* = 5) cells. Data of independent experiments are represented as the surviving fraction ± SEM. Statistical test: One way ANOVA. **B** Relative LysoTracker MFI signal measured by flow cytometry 1 h post-RT in CT26.WT (*n* = 4), HT1080 (*n* = 3), 42-MG-BA (*n* = 3) and HCT116 (*n* = 4) cells to evaluate lysosomal membrane permeabilization. Data of independent experiments are represented as the MFI ± SEM. Statistical test: One way ANOVA. **C** Fluorescent microscopy representative images of Cathepsin D signal in 42-MG-BA cells 6h post-RT, at the indicated condition. Scale bar, 10µm. **D** Scatter dot plot of Cathepsin D signal intensity measured in 42-MG-BA cells at the indicated condition, from three independents experiments. Each dot represents one cell intensity. “n” on figures indicates number of cells analyzed. Statistical test: Kruskal–Wallis test. Bar, median. **E** Relative BODIPY MFI signal measured by flow cytometry 24 h post-RT in CT26.WT, HT1080, 42-MG-BA and HCT116 cells measured, to evaluate lipid peroxidation. Data of independent experiments (*n* = 3) are represented as MFI ± SEM. Statistical test: One way ANOVA. For all Figures: *, *p* < 0.05; **, *p* < 0.01; ***, *p* < 0.001; ns, non-significant

To confirm the efficacy of NBTXR3 in destroying tumor cells, we first conducted clonogenicity tests on CT26.WT, HT1080, 42-MG-BA cells and HCT116 cells (Fig. 8A). Consistent with previous studies [12, 14–16, 18–22], these experiments emphasize the fact that NBTXR3 alone has no toxic effect. On the other hand, once activated by RT, the nanoparticles significantly enhance the destruction of tumor cells in both tested cell lines compared to RT alone. Given the abundant presence of NBTXR3 in lysosomes, we aimed to determine the impact of NBTXR3 + RT on these organelles. Specifically, we hypothesized that the radioenhancement capabilities of RT-activated NBTXR3 should result in intensified lysosomal damage, potentially triggering a greater induction of lysosomal membrane permeabilization (LMP) and subsequent release of lysosomal content into the cytosol. To test this hypothesis, we measured the LysoTracker signal in cells treated with or without NBTXR3, followed or not by irradiation (Fig. 8B). For all cell lines, no decrease in the LysoTracker signal in cells receiving only RT was observed, indicating that no LMP occurred. In stark contrast, cells treated with NBTXR3 + RT showed a significant decrease in the LysoTracker signal in all tested cell lines. Compared to the NBTXR3 alone condition, this decrease measured with NBTXR3 + RT represents around 22.24%, 16.9%, 14.9% and 26.42% of the LysoTracker signal for CT26.WT, HT1080, 42-MG-BA, and HCT116, respectively. In terms of LysoTracker signal intensity, it is interesting to note that this loss of LysoTracker signal is equivalent to approximately 56.1%, 105%, 100.6% and 180.6% of the baseline LysoTracker signal for CT26.WT, HT1080, 42-MG-BA, and HCT116, respectively.

To confirm the LMP effect induced by NBTXR3 + RT, we performed immunofluorescence experiments to assess the release of cathepsin D, a lysosomal acid hydrolase (Fig. 8C, D). Figure 8C shows a marked decrease in intensity of cathepsin D staining and appearance of a diffuse cytoplasmic signal of the cathepsin D staining in NBTXR3 + RT 42-MG-BA treated cells compared to cells treated with NBTXR3 only. In contrast, the profile of cells treated with RT only appears very similar to control cells. The lysosomal release of cathepsin D in cells treated by NBTXR3 + RT was further confirmed by measuring fluorescence intensity values in individual cells (Fig. 8D). This analysis shows a significant decrease in the signal compared to cells treated with NBTXR3 alone. It is also worth noting that no significant difference was observed between control cells and those treated with RT alone, confirming the absence of LMP induction.

A study by Gao et al. showed that ferroptosis is a lysosomal cell death process [39]. As lipid peroxidation is a hallmark of ferroptosis [40], we wanted to determine the impact of NBTXR3 + RT on this parameter by measuring the fluorescence intensity of BODIPY (Fig. 8F). The analyses reveal a notable increase in lipid peroxidation for the cell lines treated with RT alone. These results were expected, as lipid peroxidation induced by RT has been reported [41]. Interestingly, however, the levels of lipid peroxidation induced by NBTXR3 + RT is significantly higher in most cell lines tested, compared to RT alone. For CT26.WT, HT1080, 42-MG-BA and HCT116 cells, there is an increase in lipid peroxidation with NBTXR3 + RT of approximately 39%, 33%, 36% and 39% respectively, compared to RT alone.

## Discussion

The effectiveness of radiotherapy (RT) in eliminating tumor cells was demonstrated many decades ago. However, the adverse effects caused by ionizing radiation restrict its application. NBTXR3, as a first-in-class radioenhancer, has been developed to unlock the therapeutic potential of RT within cells. The primary mode of action of NBTXR3 is to enhance the effects of radiotherapy by generating reactive oxygen species (ROS), while avoiding the generation of additional side effects in patients [9, 10, 38, 42]. The efficacy of NBTXR3 combined with RT in destroying cancer cells and effectively controlling tumors has been established through investigations conducted in preclinical models [12, 14–16, 18] and human [10] studies. In addition to their enhanced ability to destroy cells, we and other teams have shown that these crystalline hafnium oxide nanoparticles possess immunomodulatory properties capable of inducing an abscopal effect [18–22]. While it is important to investigate and comprehend the biological effects mentioned above to better understand the benefits of NBTXR3 in treating solid tumors, it is important to note that these effects represent the visible outcomes of preceding biological events. Currently, our knowledge regarding these initial events is limited, apart from those related to DNA damage [16]. However, the increase in DNA damage induced by NBTXR3 + RT is also the result of earlier cellular events instigated by these nanoparticles. The aim of this article was to investigate the initial biological effects induced by NBTXR3 + RT, which could potentially elucidate its effectiveness and the cascade of resulting events previously reported.

The initial phase of our investigations involved examining the uptake of NBTXR3 nanoparticles by cancer cells and determining if it varied under different conditions, such as cell type or in vitro versus in vivo but also after the release of nanoparticles from dead cells. In vitro studies have extensively documented the capacity of NBTXR3, along with other types of nanoparticles like AuNP [43, 44], to be internalized by cancer cells, suggesting that this phenomenon is widely shared. To date, the presence of NBTXR3 in the cytoplasm 24 h after its addition onto cells has been observed in 17 distinct cell lines, including the CT26.WT cells presented in this article [12, 14, 15, 20]. In vitro findings reported in this study indicate that NBTXR3 can be internalized through both the clathrin-dependent pathway and macropinocytosis. Nonetheless, the involvement of other endocytic pathways, such as caveolin-dependent, clathrin- and caveolin-independent endocytosis, cannot be excluded due to their undetectability using the conventional TEM approach employed in our research. However, based on our TEM analyses, we postulate that the predominant entry route employed by cancer cells for the uptake of NBTXR3 nanoparticles is macropinocytosis. Macropinocytosis is a non-specific phagocytic process in which cells engulf significant amounts of extracellular fluid and particles. This mechanism is consistent with the presence of clusters observed in the cytoplasm of the three cell lines tested from the onset of the experimental kinetics, as well as the detection of NBTXR3-containing multivesicular bodies [45]. In vivo, TEM analysis on CT26.WT cells isolated from tumors previously treated with intratumoral injection of NBTXR3 reveals the formation of nanoparticle clusters that are similar to those observed in vitro, strongly suggesting that the mechanisms underlying NBTXR3 uptake are consistent between in vitro and in vivo settings. These findings align with the research conducted by Maggiorella et al. on tumor sections from HCT116 cells [12]. However, a question remains: what happens to these nanoparticles following cell destruction? When irradiated, tumor cells that have taken up NBTXR3 will undergo cell death, leading to the release of their intracellular contents. The released NBTXR3 nanoparticles will be mixed with various biological components of dead cells and may become trapped in cellular debris, potentially impeding their uptake by surviving tumor cells. Our in vitro investigation has demonstrated that nanoparticles released from dying cells can be recaptured by viable tumor cells. TEM analysis reveals that untreated 42-MG-BA-ACTB-GFP cells (not irradiated and not exposed to NBTXR3^RED^), when co-cultured with dead/dying cells previously treated with NBTXR3^RED^, are capable of internalizing released NBTXR3^RED^ nanoparticles and forming clusters similar to those previously observed in vitro and in vivo. These findings carry significant clinical implications. Indeed, these observations suggest that in patients, NBTXR3 nanoparticles released by dead cells can be endocytosed by surviving tumor cells. Whether or not these surviving cancer cells already contain NBTXR3, this uptake process will increase the intracellular concentration of NBTXR3, rendering them more immunogenic and sensitive to destruction during subsequent radiotherapy sessions [16–18]. This sets the stage for a "virtuous cycle" where the enhancement of cancer cell destruction by NBTXR3 + RT and the recapture of nanoparticles occur in succession during radiotherapy sessions. This phenomenon could also, at least in part, explain the remarkable persistence of these nanoparticles within tumor tissues and their great efficacy.

Furthermore, it is fascinating to highlight that, for the very first time, we provide evidence that these nanoparticles, upon endocytosis, exhibit an extensive accumulation within the lysosomes. These organelles serve as vital intracellular hubs responsible for the degradation of macromolecules. They play a pivotal role in maintaining calcium homeostasis and have also emerged as critical signaling centers governing the cellular response to nutrient availability. Preserving the integrity and functionality of lysosomes is vital for cellular equilibrium and overall stability. Under diverse stress conditions, the lysosomal membrane can undergo permeabilization (LMP), leading to the release of lysosomal lumen components, including cathepsins, to the cytosol, thereby triggering lysosomal-dependent cell death. In addition, the results published by Torri et al. indicate a role of lysosome-dependent ROS generation in the execution of ferroptosis [46]. In a more mechanistic point of view, our investigations unveil, for the very first time, the remarkable impact of NBTXR3 + RT on LMP, while RT alone had no effect. In our experimental conditions, this unique ability of NBTXR3 clearly demonstrates that these nanoparticles not only enhance biological responses triggered by RT but can also generate specific biological effects not achieved with RT alone.

Multiple studies have recently established a strong association between LMP and ferroptosis, a regulated form of cell death characterized by the accumulation of lipid peroxidation [47]. It has been demonstrated that radiotherapy (RT) can induce lipid peroxidation, thereby promoting ferroptosis [7, 41]. The findings presented in this article demonstrate that the level of lipid peroxidation measured with NBTXR3 + RT is significantly higher than that achieved with RT alone, suggesting that the use of these nanoparticles could enhance cell death through ferroptosis.

In a recent study, Wiernicki et al. [48] suggested that ferroptosis may negatively impact the induction of the antitumor immune response at the dendritic cell level. However, the scientific community is increasingly interested in ferroptosis as emerging evidence indicates that this programmed cell death could indirectly activate the immune system within tumor cells, thereby promoting tumor destruction [7, 49–55]. For instance, HMGB1 has been identified as a ferroptosis-related DAMP [56]. Consequently, the mechanism by which ferroptotic cells trigger potent immune responses may share similarities with traditional immunogenic cell death (ICD). Interestingly, we recently reported that NBTXR3 + RT is capable of inducing ICD [17]. Specifically, we observed an increase in HMGB1 release for HCT116, 42-MG-BA, and PANC-1 cell lines treated with NBTXR3 + RT, compared to RT alone. Thus, the results presented in this article suggest that the immunomodulatory effects of NBTXR3 + RT, as demonstrated in our previous studies, could be linked, at least in part, to LMP and ferroptosis via lipid peroxidation. Kuang et al. [57] have reported in human pancreatic cancer cells that iron-dependent LMP initiates the transfer of cathepsin B (CTSB) from the lysosome to the nucleus in the context of ferroptosis. In terms of the mechanism, the presence of CTSB within the nucleus results in DNA damage and the subsequent triggering of the stimulator of interferon response cGAMP interactor 1 (STING1/STING)-dependent DNA sensor pathway. This sequence of events ultimately culminates in autophagy-driven ferroptosis. This same phenomenon could potentially also occur in cells treated with NBTXR3 + RT, and it could, at least in part, explain the increased activity of the cGAS-STING pathway that we previously observed in HCT116-DUAL cells [16]. However, additional experiments need to be conducted to confirm this possibility.

**Fig. 9:**
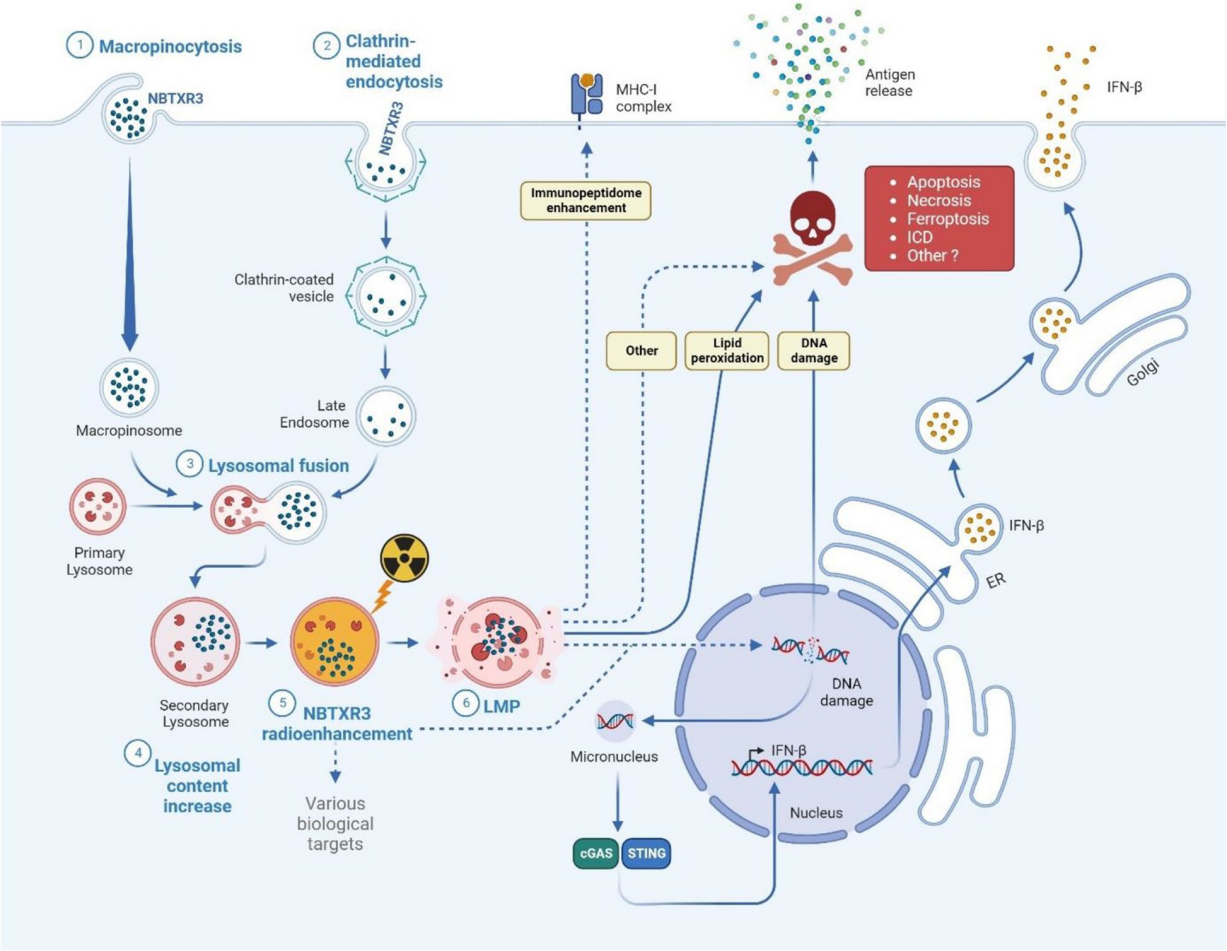
Model presenting the biological responses induced by NBTXR3 alone or activated by radiotherapy. The dotted lines indicate the potential biological pathways impacted by NBTXR3 + RT to be identified or for which a connection between two measured events previously published remains to be established

Figure 9 summarizes the biological effects highlighted in this article as well as those previously reported. However, we believe that the biological outcomes induced by NBTXR3 + RT are undoubtedly more complex and warrant further investigations. For example, Lei et al. [7] have shown that perturbing ferroptosis does not affect DNA damage and repair induced by ionizing radiation. However, we previously demonstrated that NBTXR3 + RT was capable of increasing DNA double-strand breaks and micronuclei formation [16]. Based on the work of Villagomez-Bernabe B. and Currell F. J. showing the importance of gold nanoparticles clusters for radioenhancement and the distance up to which their radioenhancement could be efficient [58], we postulate that the same properties could play an important role in the efficacy of RT-activated NBTXR3 clusters, inducing damage beyond the lysosomes. In particular, the radioenhancement capacity of NBTXR3 clusters detected close to the nucleus could cause supplementary DNA damage. Beyond this, it implies that NBTXR3 + RT can certainly generate or induce other cellular responses/mechanisms.

## Conclusion

This article illustrate the capacity of NBTXR3 to be endocytosed by cancer cells, both at the initial uptake and after release from dead cells, but also the early biological responses produced by RT-activated NBTXR3. Combined with our previous work, these data collectively demonstrate that NBTXR3 has the potential to treat any type of solid tumors. Our results have also revealed the singular biological impact of NBTXR3 + RT on the LMP. This marks for the first time that NBTXR3's radioenhancer capacity extends beyond mere amplification of RT-induced biological effects. Taken together, the universality of biological responses before and after RT make NBTXR3 a powerful partner of RT, capable of enhancing therapeutic potential of RT beyond the current limits.

### Supplementary Information

**Additional file 1: Supplemental Fig. S1.** Kinetic of NBTXR3 nanoparticles endocytosis by various cancer cells. Transmission electronic microscopy (TEM) representative images of NBTXR3 nanoparticles uptake by macropinocytosis and clathrin-mediated endocytosis in CT26.WT (upper panel), HT1080 (middle panel) and 42-MG-BA (lower panel) at 1 h and/or 3 h after addition of 400 µM NBTXR3. Membrane ruffles observed indicate membrane perturbations typical of macropinocytosis. Multivesicular bodies (MVBs) are intracellular endosomal organelles characterized by multiple internal vesicles that are enclosed within a single outer membrane, could be observed for 42-MG-BA and HT1080 cell at 3 h. White arrow: membrane ruffles enclosing a small NP aggregate; black arrow: internalized NBTXR3 nanoparticles cluster in early endosomes; blue arrow: multivesicular bodies. Scale bar, 300 nm. Abbreviations: Cyt, cytoplasm; Nuc, nucleus. **Supplemental Fig. S2.** Additional cell lines for granularity analysis. Following addition of 400 µM NBTXR3, the evolution of the cellular granularity at the indicated time-points was carried out by flow cytometry for the MDA-MB-231 and THP-1 cells. A Representative flow cytometry analysis of the evolution of the cellular granularity over time. B Kinetic of cellular granularity profile evolution for the MDA-MB-231 (*n*=3) and THP-1 (*n*=4) cells. Data of independent experiments are represented as the relative granularity to CTL±SEM. Statistical test: Paired t-test. *, *p*<0.05. C Baseline granularity of the tested cell lines. Data of independent experiments are represented as the mean granularity (SSC-A)±SEM. D Comparison of granularity profile evolution for tested cell lines. Data of independent experiments are represented as the relative granularity to CTL±SEM. **Supplemental Fig. S3.** Validation tests for fluorescent labeling of NBTXR3. Validation tests were carried out both on the labeling of NBTXR3 with dextran-tetramethylrhodamine 70kDa (#D1818, upper panel) and 3kDa (#D3308, lower panel). Solutions with 400µM of NBTXR3 or NBTXR3-dextran labeled with a fluorescence marker were placed in µ-Dish petri dishes from Ibidi. To check the stability of NBTXR3-dextran labeling under lysosomal conditions (pH 4), we added 1M hydrochloric acid to the medium. The solutions were then incubated at 37°C for 15 minutes. We also prepared a dextran-only solution by diluting dextran 1:100 (#D1818) or 1:500 (#D3308) in distilled water. The volume of dextran solution used in the petri dishes, both for pure Dextran and the wash dextran (obtained during the final centrifugation step in NBTXR3-dextran preparation), was the same as that for NBTXR3. These solutions were incubated at 37°C for 1 hour. Image acquisition was done with a fluorescent microscope. **Supplemental Fig. S4.** Representative evolution of LysoTracker signal over time analyzed by fluorescent microscopy for CT26.WT (upper panel), HT1080 (middle panel), and HCT116 cells (lower panel). **Supplemental Fig. S5.** Additional cell lines for LysoTracker signal analysis. Following addition of 400 µM NBTXR3, the evolution LysoTracker signal at the indicated time-points was carried out by flow cytometry for the MDA-MB-231, and THP-1 cells. A Representative evolution of LysoTracker signal over time analyzed by flow cytometry. Kinetic modification of LysoTracker profile for B MDA-MB-231 (*n*=3), and C THP-1 (*n*=4) cells. Data of independent experiments are represented as the relative LysoTracker MFI to CTL±SEM. Statistical test: Paired t-test. *, *p*<0.05; **, *p*<0.01; D. Comparison of MFI LysoTracker profile evolution for tested cell lines. Data of independent experiments are represented as the relative MFI to CTL±SEM. **Supplemental Fig. S6.** Additional representative TEM images of NBTXR3RED nanoparticles uptake by 42-MG-BA-ACTB-GFP cells 48h after co-culture with dead 42-MG-BA NBTXR3RED cells. Scale bar: 0.5µm. Abbreviations: Cyt, cytoplasm; MVB, multivesicular body. **Supplemental Table 1.** List of lysosome-associated genes (GO:0005764) significantly modulated by NBTXR3 treatment vs. untreated cells (CTL), 24 h after treatment with NBTXR3 in CT26.WT cells, analyzed by RNAseq.

## Data Availability

All data generated or analyzed during this study are included in this published article [and its supplementary information files].

## Abbreviations

ICD: Immunogenic Cell Death
IR: Ionizing radiation
LMP: Lysosomal Membrane Permeabilization
MFI: Mean Fluorescence Intensity
ROS: Reactive Oxygen Species
RT: Radiotherapy
TEM: Transmission Electron Microscopy
