# Supplementary material for: Radiotherapy-activated NBTXR3 nanoparticles promote ferroptosis through induction of lysosomal membrane permeabilization
Source: J Exp Clin Cancer Res. 2024 Jan 3;43:11. doi: 10.1186/s13046-023-02938-0 (PMC10762921; doi:10.1186/s13046-023-02938-0)
Supplement: Supplementary file 1 — Additional file 1: Supplemental Fig. S1. Kinetic of NBTXR3 nanoparticles endocytosis by various cancer cells. Transmission electronic microscopy (TEM) representative images of NBTXR3 nanoparticles uptake by macropinocytosis and clathrin-mediated endocytosis in CT26.WT (upper panel), HT1080 (middle panel) and 42-MG-BA (lower panel) at 1 h and/or 3 h after addition of 400 µM NBTXR3. Membrane ruffles observed indicate membrane perturbations typical of macropinocytosis. Multivesicular bodies (MVBs) are intracellular endosomal organelles characterized by multiple internal vesicles that are enclosed within a single outer membrane, could be observed for 42-MG-BA and HT1080 cell at 3 h. White arrow: membrane ruffles enclosing a small NP aggregate; black arrow: internalized NBTXR3 nanoparticles cluster in early endosomes; blue arrow: multivesicular bodies. Scale bar, 300 nm. Abbreviations: Cyt, cytoplasm; Nuc, nucleus. Supplemental Fig. S2. Additional cell lines for granularity analysis. Following addition of 400 µM NBTXR3, the evolution of the cellular granularity at the indicated time-points was carried out by flow cytometry for the MDA-MB-231 and THP-1 cells. A Representative flow cytometry analysis of the evolution of the cellular granularity over time. B Kinetic of cellular granularity profile evolution for the MDA-MB-231 (n=3) and THP-1 (n=4) cells. Data of independent experiments are represented as the relative granularity to CTL±SEM. Statistical test: Paired t-test. *, p<0.05. C Baseline granularity of the tested cell lines. Data of independent experiments are represented as the mean granularity (SSC-A)±SEM. D Comparison of granularity profile evolution for tested cell lines. Data of independent experiments are represented as the relative granularity to CTL±SEM. Supplemental Fig. S3. Validation tests for fluorescent labeling of NBTXR3. Validation tests were carried out both on the labeling of NBTXR3 with dextran-tetramethylrhodamine 70kDa (#D1818, upper panel) and 3k [file 13046_2023_2938_MOESM1_ESM.docx]

# Supplemental data


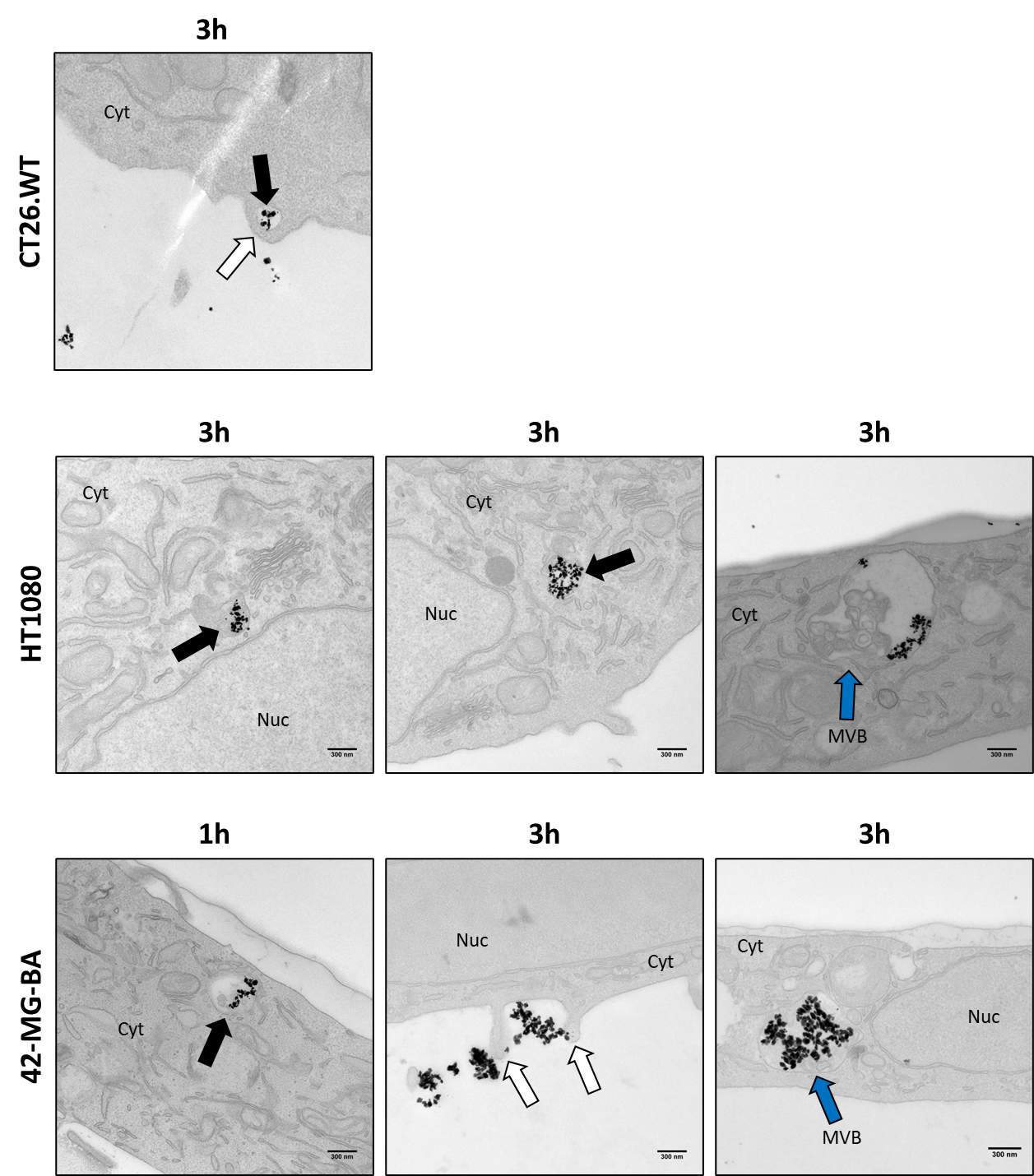


**Supplemental Fig. S1.** **Kinetic of NBTXR3 nanoparticles endocytosis by various cancer cells.** Transmission electronic microscopy (TEM) representative images of NBTXR3 nanoparticles uptake by macropinocytosis and clathrin-mediated endocytosis in CT26.WT (upper panel), HT1080 (middle panel) and 42-MG-BA (lower panel) at 1 h and/or 3 h after addition of 400 µM NBTXR3. Membrane ruffles observed indicate membrane perturbations typical of macropinocytosis. Multivesicular bodies (MVBs) are intracellular endosomal organelles characterized by multiple internal vesicles that are enclosed within a single outer membrane, could be observed for 42-MG-BA and HT1080 cell at 3 h. White arrow: membrane ruffles enclosing a small NP aggregate; black arrow: internalized NBTXR3 nanoparticles cluster in early endosomes; blue arrow: multivesicular bodies. Scale bar, 300 nm. Abbreviations: Cyt, cytoplasm; Nuc, nucleus.

**
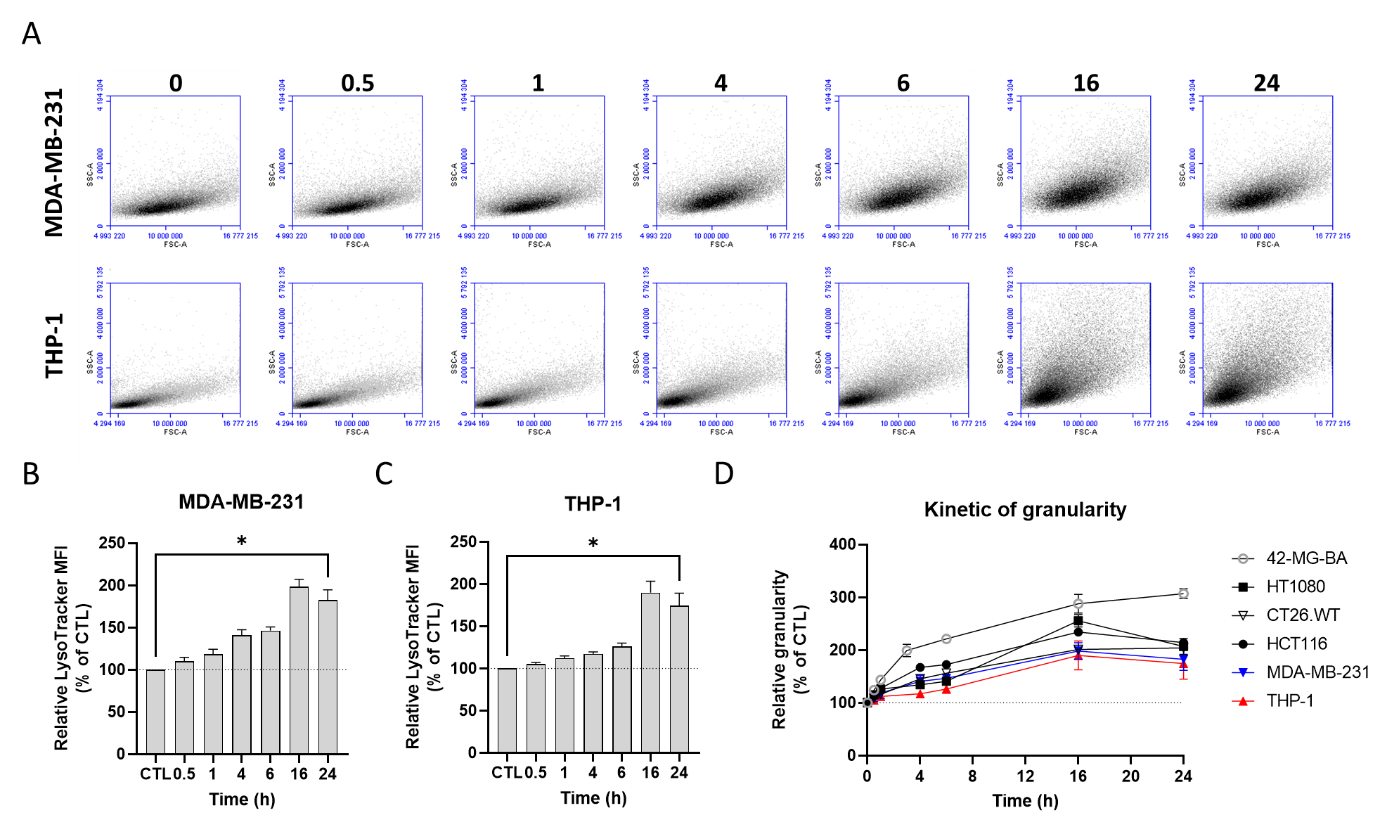
**

**Supplemental Fig. S2.** **Additional cell lines for granularity analysis.** Following addition of 400 µM NBTXR3, the evolution of the cellular granularity at the indicated time-points was carried out by flow cytometry for the MDA-MB-231 and THP-1 cells. **A** Representative flow cytometry analysis of the evolution of the cellular granularity over time. **B** Kinetic of cellular granularity profile evolution for the MDA-MB-231 (n=3) and THP-1 (n=4) cells. Data of independent experiments are represented as the relative granularity to CTL±SEM. Statistical test: Paired t-test. *, p<0.05. **C** Baseline granularity of the tested cell lines. Data of independent experiments are represented as the mean granularity (SSC-A)±SEM. **D** Comparison of granularity profile evolution for tested cell lines. Data of independent experiments are represented as the relative granularity to CTL±SEM.

**
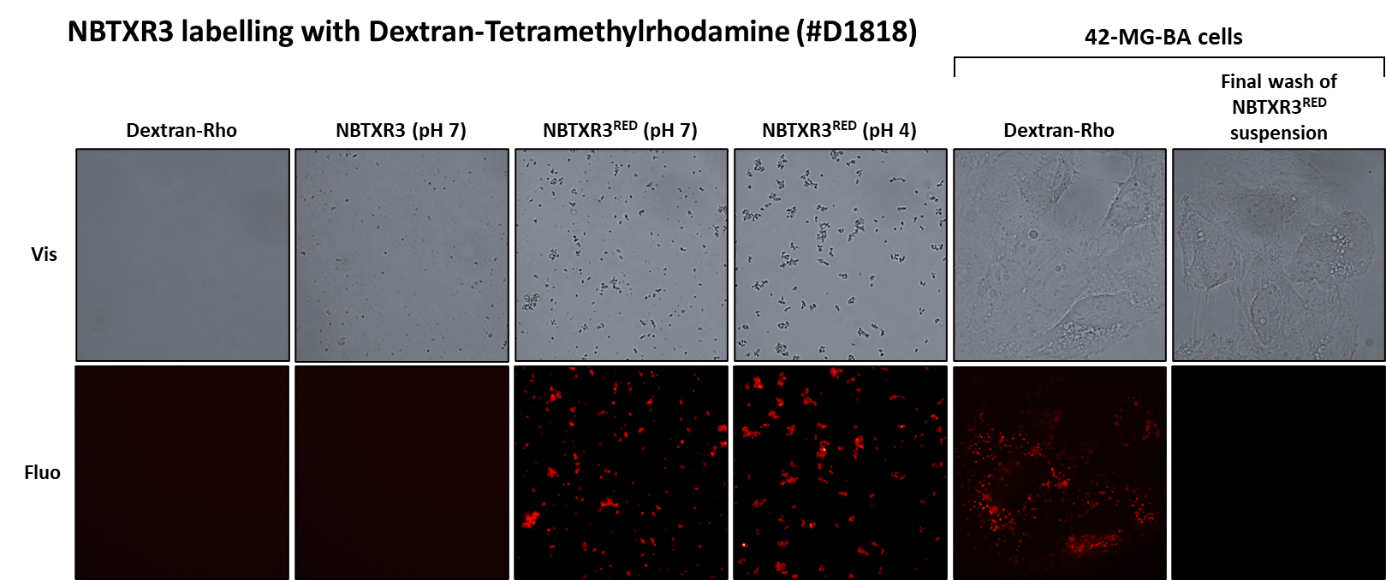
**

**
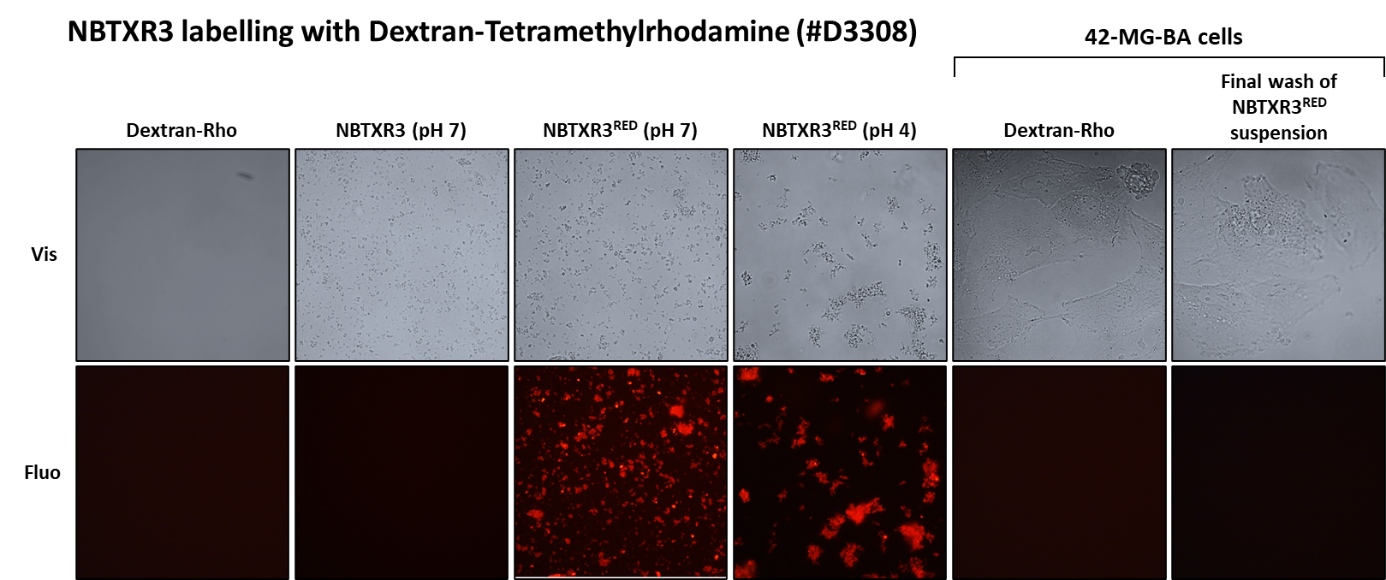
**

**Supplemental Fig. S3.** **Validation tests for fluorescent labeling of NBTXR3.** Validation tests were carried out both on the labeling of NBTXR3 with dextran-tetramethylrhodamine 70kDa (#D1818, upper panel) and 3kDa (#D3308, lower panel). Solutions with 400µM of NBTXR3 or NBTXR3-dextran labeled with a fluorescence marker were placed in µ-Dish petri dishes from Ibidi. To check the stability of NBTXR3-dextran labeling under lysosomal conditions (pH 4), we added 1M hydrochloric acid to the medium. The solutions were then incubated at 37°C for 15 minutes. We also prepared a dextran-only solution by diluting dextran 1:100 (#D1818) or 1:500 (#D3308) in distilled water. The volume of dextran solution used in the petri dishes, both for pure Dextran and the wash dextran (obtained during the final centrifugation step in NBTXR3-dextran preparation), was the same as that for NBTXR3. These solutions were incubated at 37°C for 1 hour. Image acquisition was done with a fluorescent microscope.

**
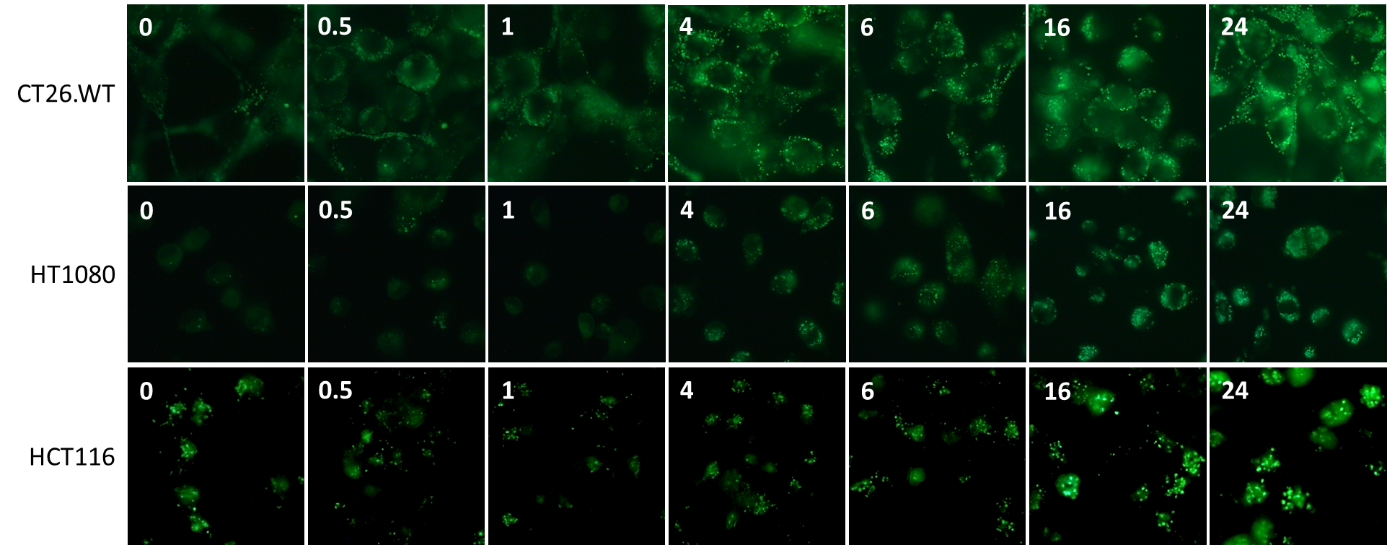
**

**Supplemental Fig. S4.** Representative evolution of LysoTracker signal over time analyzed by fluorescent microscopy for CT26.WT (upper panel), HT1080 (middle panel), and HCT116 cells (lower panel).

**
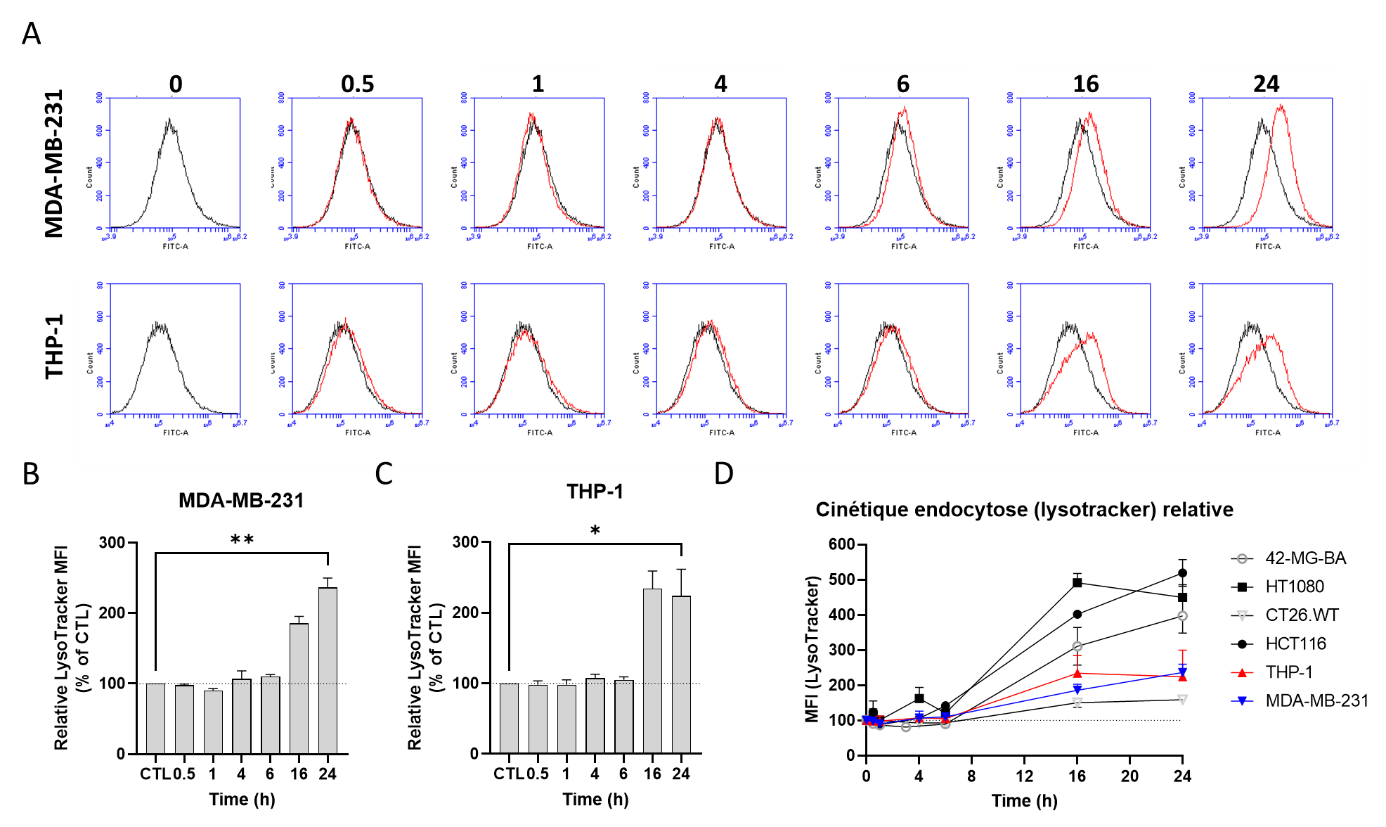
**

**Supplemental Fig. S5.** **Additional cell lines for LysoTracker signal analysis.** Following addition of 400 µM NBTXR3, the evolution LysoTracker signal at the indicated time-points was carried out by flow cytometry for the MDA-MB-231, and THP-1 cells. **A** Representative evolution of LysoTracker signal over time analyzed by flow cytometry. Kinetic modification of LysoTracker profile for **B** MDA-MB-231 (n=3), and **C** THP-1 (n=4) cells. Data of independent experiments are represented as the relative LysoTracker MFI to CTL±SEM. Statistical test: Paired t-test. *, *p*<0.05; **, *p*<0.01; **D**. Comparison of MFI LysoTracker profile evolution for tested cell lines. Data of independent experiments are represented as the relative MFI to CTL±SEM.

**
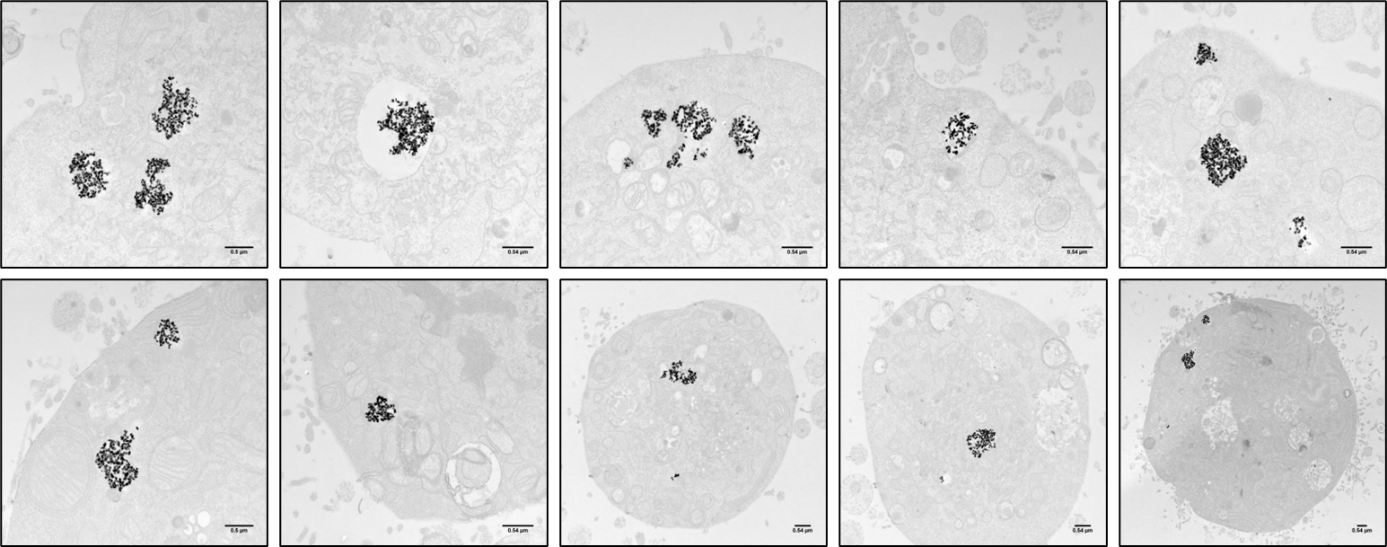
**

**Supplemental Fig. S6.** Additional representative TEM images of NBTXR3^RED^ nanoparticles uptake by 42-MG-BA-ACTB-GFP cells 48h after co-culture with dead 42-MG-BA NBTXR3^RED^ cells. Scale bar: 0.5µm. Abbreviations: Cyt, cytoplasm; MVB, multivesicular body.

**Supplemental Table 1.** List of lysosome-associated genes (GO:0005764) significantly modulated by NBTXR3 treatment vs. untreated cells (CTL), 24 h after treatment with NBTXR3 in CT26.WT cells, analyzed by RNAseq.

| Genes_symbol | ENSEMBL | padjBH_CTL_vs_NBTXR3_24h | LogFC_CTL_vs_NBTXR3_24h |
| --- | --- | --- | --- |
| DPP7 | ENSMUSG00000026958 | 4,20E-03 | 1.20e+00 |
| NEU1 | ENSMUSG00000007038 | 4,20E-03 | 1.01e+00 |
| CTNS | ENSMUSG00000005949 | 8,88E-03 | 8.92e-01 |
| FUCA2 | ENSMUSG00000019810 | 8,88E-03 | 8.29e-01 |
| SLC66A1 | ENSMUSG00000028744 | 1,05E-02 | 1.02e+00 |
| DNASE2A | ENSMUSG00000003812 | 1,11E-02 | 1.03e+00 |
| LAMP2 | ENSMUSG00000016534 | 1,11E-02 | 6.91e-01 |
| OSTM1 | ENSMUSG00000038280 | 1,11E-02 | 7.16e-01 |
| TPP1 | ENSMUSG00000030894 | 1,11E-02 | 7.49e-01 |
| TECPR1 | ENSMUSG00000066621 | 1,31E-02 | 8.65e-01 |
| GRN | ENSMUSG00000034708 | 1,36E-02 | 8.59e-01 |
| ABCB6 | ENSMUSG00000026198 | 1,63E-02 | 6.76e-01 |
| ATP6AP2 | ENSMUSG00000031007 | 1,63E-02 | 8.22e-01 |
| LAMP1 | ENSMUSG00000031447 | 1,63E-02 | 5.89e-01 |
| CD68 | ENSMUSG00000018774 | 1,65E-02 | 9.64e-01 |
| HYAL1 | ENSMUSG00000010051 | 1,65E-02 | 9.28e-01 |
| FLCN | ENSMUSG00000032633 | 1,69E-02 | 5.15e-01 |
| GLMP | ENSMUSG00000001418 | 1,69E-02 | 7.09e-01 |
| HEXA | ENSMUSG00000025232 | 1,83E-02 | 6.57e-01 |
| MFSD1 | ENSMUSG00000027775 | 2,88E-02 | 6.31e-01 |
| PSAP | ENSMUSG00000004207 | 2,88E-02 | 5.34e-01 |
| VPS26A | ENSMUSG00000020078 | 2,88E-02 | 5.23e-01 |
| MCOLN1 | ENSMUSG00000004567 | 3,00E-02 | 5.71e-01 |
| ATP6V0D1 | ENSMUSG00000013160 | 3,16E-02 | 5.87e-01 |
| SLC39A8 | ENSMUSG00000053897 | 3,16E-02 | -6.20e-01 |
| GBA | ENSMUSG00000028048 | 3,18E-02 | 5.92e-01 |
| STX3 | ENSMUSG00000041488 | 3,37E-02 | 5.67e-01 |
| CLN5 | ENSMUSG00000022125 | 3,72E-02 | 5.13e-01 |
| CTSA | ENSMUSG00000017760 | 3,72E-02 | 6.89e-01 |
| IDUA | ENSMUSG00000033540 | 3,76E-02 | 6.77e-01 |
| CTSD | ENSMUSG00000007891 | 3,79E-02 | 4.97e-01 |
| GAA | ENSMUSG00000025579 | 3,79E-02 | 6.12e-01 |
| SLC2A6 | ENSMUSG00000036067 | 3,79E-02 | 8.24e-01 |
| ARSA | ENSMUSG00000022620 | 4,30E-02 | 5.05e-01 |
| ASAH1 | ENSMUSG00000031591 | 4,30E-02 | 5.46e-01 |
